# A Biomimetic Fiber‐Entangled Permeable Electronic Skin for Strain‐Insensitive and High‐Resolution Tactile Sensing

**DOI:** 10.1002/advs.202512111

**Published:** 2025-08-28

**Authors:** Ruixiang Qu, Menghui Ji, Ningjing Zhou, Rongdi Zhang, Huijiao Ji, Min Zou, Huacheng He, Yu Zhang, Fuguang Chen, Mengjia Chen, Jiujiang Ji, Zhijun Ma

**Affiliations:** ^1^ Research Center for New Materials Computing Zhejiang Lab Hangzhou 311100 China; ^2^ Research Center for Computing Sensing Zhejiang Lab Hangzhou 311100 China; ^3^ School of Materials Science and Engineering Zhejiang University Hangzhou 310058 China; ^4^ Oujiang Laboratory Wenzhou 325000 China; ^5^ Department of Pharmacology School of Pharmacy Southwest Medical University Luzhou 646000 China; ^6^ Department of Chemistry Tsinghua University Beijing ‌100084‌ China

**Keywords:** electronic skins, high‐resolution, pressure imaging, strain‐insensitive, wearable devices

## Abstract

Electronic skins (e‐skins) incorporating island architectures represent a promising platform for strain‐insensitive tactile sensing by mechanically decoupling sensing units from deformations. However, conventional island designs encounter stress concentration issues caused by inherent modulus mismatches, critically limiting achievable island densities. This limitation forces a stubborn trade‐off between strain‐insensitivity and sensing resolution. Here, inspired by the entangled elastin networks surrounding human tactile receptors, a biomimetic fiber‐entangled island architecture is proposed that addresses the stress concentration issue, providing a viable solution for strain‐insensitive and high‐resolution tactile sensing. The mechanism by which the fiber‐entangled architecture mitigates stress concentration is based on the strain‐dependent reorientation of its constituent fibers. As a demonstration of this solution, a pressure sensing e‐skin exhibiting simultaneous high resolution (100 unit cm^−2^) and low strain interference (gauge factor < 0.03) is developed. Implemented with artificial neural networks, the e‐skin demonstrates proof‐of‐concept functionality as a wearable Braille point‐to‐read system. The fiber‐entangled architecture proposed here will emerge as a versatile platform for next‐generation humanoid sensing.

## Graphical abstract

A biomimetic fiber‐entangled island architecture is proposed to address the stress concentration issues commonly observed in conventional island arrays. Based on this architecture, a pressure‐sensing e‐skin with simultaneously high spatial resolution, low strain interference, and excellent permeability is developed, which is further applied to wearable pressure imaging.

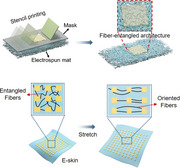

## 1 Introduction

Human skin perceives the surrounding environment through exquisite tactile interactions, particularly pressure sensing mediated by densely distributed tactile receptors in the dermis (50 to 240 units cm^−2^).^[^ ^1^, ^2^, ^3^ ^]^ These receptors are insensitive to stretch, enabling the neural system to achieve high‐fidelity tactile cognition during complex tasks such as dexterous manipulation and locomotion.^[^ ^4^, ^5^ ^]^ To emulate this somatosensory capability, intensive endeavors have been devoted to developing stretchable electronic skins  (e‐skins) featuring high‐density sensitive units resembling dermal tactile receptors.^[^ ^6^, ^7^, ^8^, ^9^ ^]^ State‐of‐the‐art devices have achieved sensor densities of thousands of units per square centimeter, and are likely to boost the emergence of artificial haptics, which would be a new era of tactile repair and tactile enhancement.^[^ ^10^, ^11^, ^12^, ^13^ ^]^ However, unlike human skin, these synthetic systems suffer significant performance degradation under mechanical strain. This limitation arises from the inherent coupling of mechanical deformations across different directions within individual sensing units, resulting in severe stretching crosstalk that considerably compromises sensing reliability.^[^ ^14^, ^15^ ^]^

To decouple the stretching crosstalk from tactile sensing, various strategies have been proposed to isolate sensing elements from applied deformations. These approaches primarily employ structural designs that incorporate rigid sensing islands within stretchable polymer substrates.^[^ ^16^, ^17^, ^18^, ^19^ ^]^ These rigid islands possess significantly higher elastic moduli than the substrates and are therefore barely stretched. Nevertheless, due to the modulus mismatch between the islands and substrate, tensile stress tends to concentrate at the island‐substrate interface, making it susceptible to interfacial cracks under stretching. This ultimately results in crack propagation and device failure.^[^ ^20^, ^21^, ^22^ ^]^ Crucially, the stress concentration intensifies with increasing island density, which severely restricts the attainable pixel density of the island‐structured e‐skins, making them 2‐3 orders of magnitude inferior to that of the human skin.^[^ ^23^, ^24^ ^]^ To date, e‐skins that can simultaneously achieve low strain interference and high pixel density remain elusive, despite their critical importance for artificial haptics.^[^ ^25^, ^26^, ^27^ ^]^

**Figure 1:**
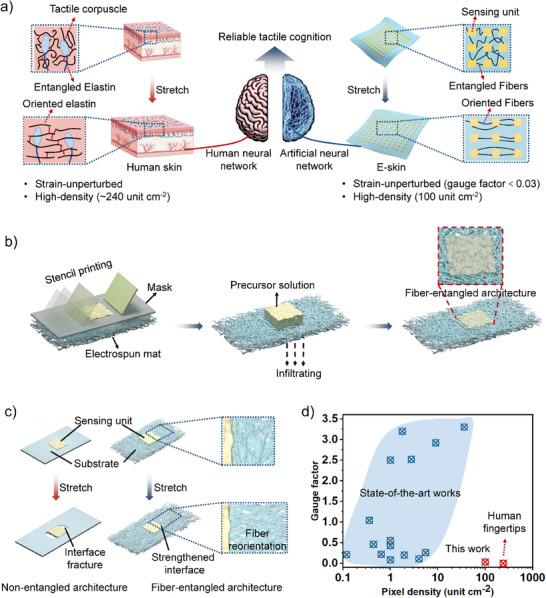
Fiber‐entangled island structure. a) Schematic illustration of a humanoid sensory neural pathway based on the fiber‐entangled structure. The e‐skin is characterized by low stretch‐crosstalk and high array density. b) Schematic diagrams showing the stencil printing of the fiber‐entangled structure. c) Schematic diagrams illustrating the fiber‐entangled structure overcoming the modulus mismatch issue upon stretching. d) Comparison of the gauge factor and pixel density of our e‐skin with existing stretchable and multipixel e‐skins. The relevant references are listed in Table S2 (Supporting Information).

In human skin, connective tissue has been identified as a key structural component encapsulating densely packed dermal tactile receptors.^[^ ^28^, ^29^ ^]^ Characterized by a low elastic modulus (≈0.5 MPa).^[^ ^30^ ^]^ the connective tissue is capable of isolating the tactile receptors from deformations, forming a biological island‐structured sensing array.^[^ ^31^ ^]^ Distinct from artificial island structures, the biological counterpart incorporates entangled elastin networks within the connective tissue.^[^ ^32^ ^]^ which are capable of reorient under stretch to dissipate tensile stress (**Figure** **1**a).^[^ ^33^ ^]^ This dynamic establishes human skin as an ideal island architecture featured by superior mechanical strength.^[^ ^34^ ^]^ Therefore, by mimicking the entangled elastin architecture of connective tissue, it is possible to overcome the longstanding stress concentration issue in tactile sensing, enabling e‐skins integrating high spatial resolution and minimal strain interference. Besides, permeability represents a critical attribute of e‐skin, enabling transdermal metabolism essential for maintaining physiological homeostasis during use.^[^ ^35^ ^]^ The absence of permeability raises significant biocompatibility concerns during prolonged wear, potentially leading to inflammatory responses or dermatological disorders.^[^ ^36^, ^37^ ^]^ In addition to replicating the entangled elastin architecture, achieving permeability in e‐skins deserves equal priority.^[^ ^38^, ^39^, ^40^, ^41^, ^42^ ^]^

In this work, by mimicking the elastin network architecture surrounding tactile receptors, we developed a fiber‐entangled island architecture that simultaneously enables high‐density tactile sensing and strain isolation. This design enables the fabrication of a pressure sensing e‐skin with human fingertip‐like tactile resolution (100 unit cm^−2^), low strain interference (gauge factor < 0.03), and high permeability (Figure 1a). The architecture can be simply fabricated by stencil printing a precursor solution onto an electrospun fibrous mat. Capillary‐driven infiltration anchors the precursor within the 3D fibrous matrix, forming mechanically reinforced islands with fibers penetrating through them (Figure 1b). Crucially, upon stretching, the fibers at the island‐substrate interface undergo reorientation similar to that of entangled elastin networks, effectively eliminating localized stress concentrations caused by modulus mismatch between the islands and substrate (Figure 1c). Consequently, the fiber‐entangled island array can withstand large tensile strains while isolating the islands from the mechanical deformation, even at high pixel densities. This results in an e‐skin that, in terms of pixel density and strain interference, is the closest to human fingertip skin to date (Figure 1d). Furthermore, integration of the fiber‐entangled e‐skin with artificial neural networks enables a wearable Braille point‐to‐read system, highlighting the potential of the fiber‐entangled architectures for next‐generation humanoid sensing.

## 2 Design Concept of the Fiber‐Entangled Architecture

Prior to designing the e‐skin, finite element analysis was conducted on conventional island arrays with nonporous substrates to investigate the mechanical origins of modulus mismatch (Figure S1a, Supporting Information). Simulations revealed substantial interfacial stress concentrations at island‐substrate boundaries, arising from the stiffness contrast between rigid islands and compliant substrates. The stress concentration increased quadratically with island density (Figure S1b, Supporting Information), resulting in interfacial mechanical failure of dense islands under tensile strain. This phenomenon arised inherently from the constitutive mechanics of heterogeneous materials, making it a persistent limitation that is impervious to mitigation through adjustments of structural parameters.

Here, inspired by elastin, in which stochastic amino acid entanglements dynamically reoriented under strain to dissipate mechanical stress^[^ ^43^ ^]^ we proposed an island array with outstanding mechanical stability. Central to this strategy was a fiber‐entangled island architecture, featuring a multitude of randomly oriented fibers at the island‐substrate interface (Figure 1c). The substrate was composed of a low‐modulus electrospun elastomeric mat, while the islands consisted of high‐modulus pressure‐sensitive materials. Upon stretching, interfacial fibers undergo strain‐dependent reconfiguration, mimicking the dynamic reorganizing of entangled elastin. This behavior redistributed stress throughout the fiber network, effectively suppressing interfacial crack even at high island densities. Furthermore, owing to the pronounced modulus difference between the island and substrate, stretching of the array predominantly localized strain within the compliant substrate, which was advantageous for stretch‐insensitive pressure sensing (Figure S2 (Supporting Information) and Video S1 (Supplementary Video 1)).^[^ ^44^, ^45^ ^]^

**Figure 2:**
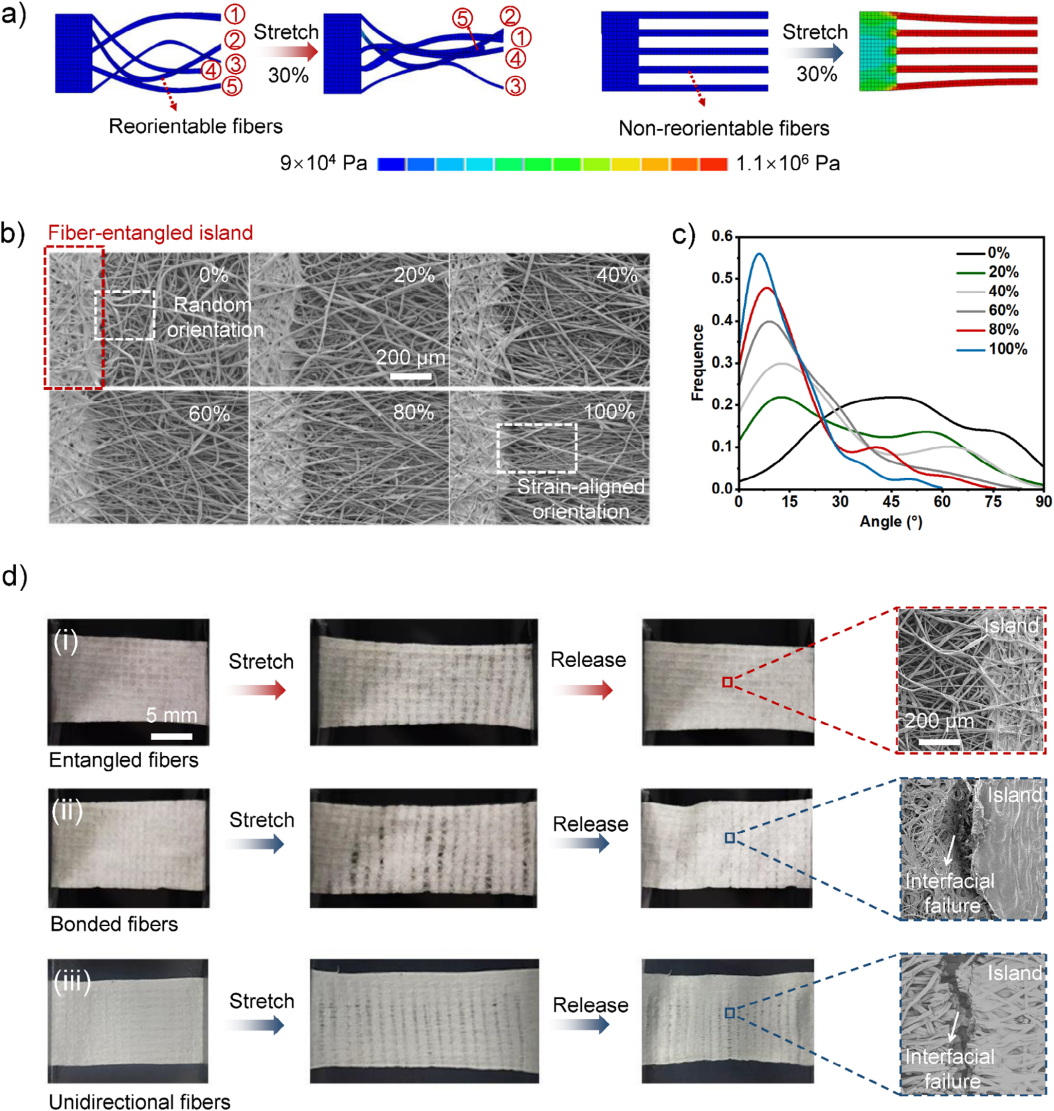
The mechanism of the fiber‐entangled structure dissipating tensile stress. a) Finite‐element simulation of the fiber behaviors under 30% strain. Left: The fibers in the fiber‐entangled structure reoriented to dissipating tensile stress upon stretching. Right: The fibers incapable of reorientation precipitate pronounced stress concentration. The elastic modulus of the fibers and rigid islands were 150 kPa and 1 GPa, respectively, with Poisson's ratios of 0.3 for both materials. b) The SEM images showing the reorientation behavior of the fiber upon stretching. c) Quantitative assessment demonstrating the fibers in the fiber‐entangled structure tended to shift from a stochastic orientation to a strain‐aligned orientation upon stretching. d) The SEM images showing a single stretch of the arrays (100 unit cm^−2^) fabricated based on differnet fibrous mats. The mat with reorientable fibers retains an intact interface. The mats with bonded fibers and quasi‐unidirectional fibers suffered from interfacial failure.

The stress‐dissipation behavior of the fiber‐entangled architecture was validated through integrated simulations and experiments. On the simulation front, simplified 2D models were developed, incorporating rigid islands interconnected by fiber (**Figure** **2**a). The simulations revealed that non‐reorientable fibers, initially aligned with the strain axis, exhibited pronounced stress concentration at 30% strain. In contrast, reorientable fibers with randomized initial orientations mitigated interfacial stress by an order of magnitude lower through reorientation. Experimentally, a fiber‐entangled island array with submillimeter‐scale islands was fabricated using stencil printing. To ensure unit‐to‐unit uniformity during printing, the viscosity of the precursor solution was precisely controlled (Figure S3, Supporting Information). The fabricated array was subjected to varying tensile strains, and fiber orientation changes at the island‐substrate interface observed (Figure 2b; Figure S4, Supporting Information). With increasing strain, the fibers progressively transitioned from a stochastic to a strain‐aligned orientation, as quantitatively verified by alignment angle dispersion analysis (Figure 2c; Figure S5, Supporting Information). These results demonstrate the feasibility of our fiber‐entangled architecture in mitigating stress concentration. More importantly, this stress‐dissipation capability remained robust regardless of fiber parameters (e.g., fiber diameter, density), as the substantial elastic modulus mismatch between the fibrous mat and rigid islands persisted across different fiber parameters, further highlighting the versatility of the fiber‐entangled architecture (Figure S6, Supporting Information).

To further elucidate the stress dissipation mechanism, fibrous mats composed of distinct fiber types were used to fabricate island arrays, and their tensile stability was analyzed comparatively. In contrast to the fiber‐entangled array, which retained interfacial integrity following stretching, the arrays fabricated from non‐reorientable fibers, including bonded fibers and quasi‐unidirectional fibers, demonstrated interfacial failures after a 50% tensile strain (Figure 2d). The quasi‐unidirectional fibers was fabraicated by drum‐assisted electrospinning,^[^ ^46^ ^]^ exhibiting nearly consistent alignment parallel to the tensile direction (Figure S7, Supporting Information), thereby eliminating the capacity for reorientation. And the bonded fibers were produced through thermocompression of a fibrous mat, where fixed fiber intersections restricted reorientation. Moreover, replacing the fibrous mat with a nonfibrous substrate yielded an array devoid of fibers, which failed prematurely due to detachment of rigid islands (Figure S8, Supporting Information). These integrated simulations and experiments conclusively demonstrate that dynamic fiber reorientation within the fiber‐entangled architecture is essential for dissipating stress concentration, rendering this architecture advantageous for fabricating e‐skins integrating high pixel density and low strain interference.

## 3 Fabrication of e‐Skin based on the Fiber‐Entangled Architecture

**Figure 3:**
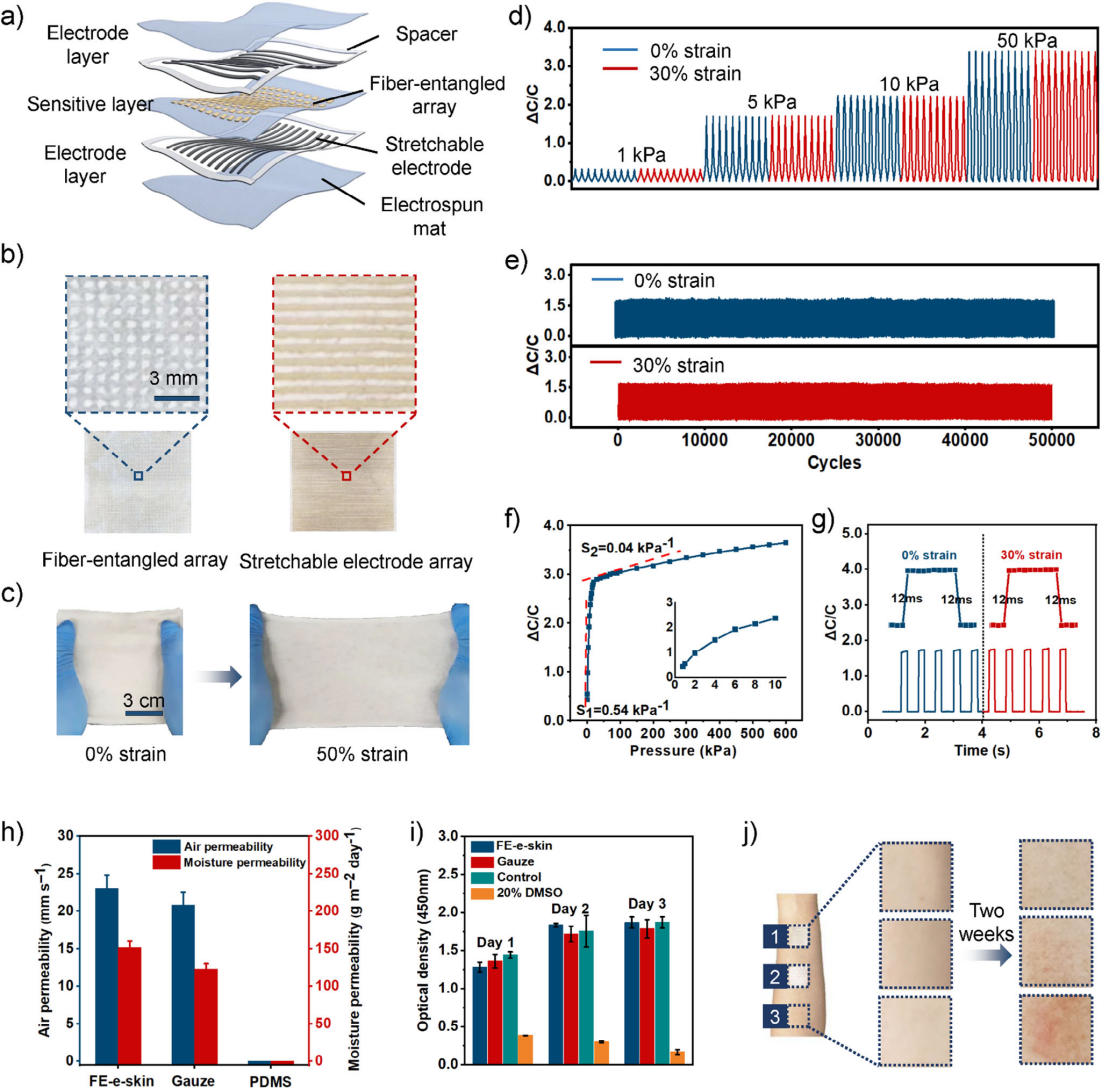
The e‐skin based on the fiber‐entangled structure. a) Schematic illustration showing the structure of the e‐skin. b) Microphotographs showing the high pixel density of the e‐skin. Left: The sensitive layer composed of fiber‐entangled array with a pixel density of 100 unit cm^−2^. Right: The electrode layer composed of parallel electrode with a line distance of 1 mm. c) Optical photographs showing the stretchability of the e‐skin. d–g) Strain‐insensitive pressure sening performances of the e‐skin under 0% and 30% strain, embodied by capacitance responses to various pressures (d), fatigue tests with pressure loading of 5 kPa (e), capacitance responses over the pressure range up to 600 kPa (f), and response time (g). h–j) High biocompatibility of the e‐skin, displayed by air permeability and moisture permeability (h), cytotoxicity test (i), and skin irritation results on the forearms of the volunteer (j). 1) FE‐e‐skin; 2) Commercial gauze; 3) impermeable PDMS flim.

As a proof of concept, a poly(styrene ethylene butylene styrene) electrospun mat (SEBS mat) and a thermopl astic polyurethane (TPU)/1‐ethyl‐3‐methylimidazolium bis(trifluoromethylsulfonyl)imide (EMIM TFSI) ionic elastomer were chosen as the substrate and islands, respectively (Figure S9, Supporting Information). Consequently, a pressure‐sensitive fiber‐entangled array was fabricated as the sensitive layer. Further integration of electrode layers (Ag/SEBS composite) and spacers (crucial for reducing strain interference^[^ ^23^ ^]^) led to the creation of a fiber‐entangled e‐skin (FE‐e‐skin) characterized by high pixel density (100 units cm^−2^) and low strain interference (**Figure** **3**a–c). The fabrication details of each layer were described in the Method Section. Tensile testing revealed that the FE‐e‐skin possessed remarkable stretchability, demonstrating a breaking elongation exceeding 900% and withstanding over 3000 stretching cycles at 50% strain (Figure S10 (Supporting Information) and Video S2 (Supplementary Video 2)). Although minor pitch variations were observed in the sensing array and electrodes after 3000 cycles, these did not compromise pressure sensing performance, as detailed in Figure S11 (Supporting Information). Furthermore, the scalability of the fabrication approach was confirmed through the production of a 10000‐unit array (Figure 3b) featuring programmable pixel densities (Figure S12, Supporting Information).

To enhance device performance, the electrodes were carefully optimized. The resulting electrode demonstrated excellent mechanical properties for stretchable e‐skin applications (Figure S13a, Supporting Information), including a low elastic modulus (≈560 kPa) and exceptional fracture elongation (>900%). Compared to commercial stretchable silver paste, the Ag/SEBS‐based electrodes demonstrated superior durability, maintaining structural integrity without morphological degradation or Ag delamination even after 3000 stretching cycles (Figure S13b–e, Supporting Information). This enhanced robustness can be attributed to two key factors: the intrinsically favorable mechanical properties of SEBS, and the embedded interfacial structure between the electrode and mat (Figure S13f, Supporting Information). Moreover, the electrodes exhibited low resistance (<15 Ω at 30% strain), minimizing the influence of electrode impedance on the sensing signal (Figure S13g, Supporting Information).

Systematic characterization was conducted by testing the pressure sensing properties of a single‐pixel FE‐e‐skin, which demonstrated the functional advantages of its fiber‐entangled architecture. The pressure‐sensing mechanism originated from an ionic elastomer‐mediated electric double layer, with detailed working principles illustrated in Figure S14 (Supporting Information). The pronounced modulus disparity between the SEBS mat and sensing units isolated sensing units from tensile strain (FigureS15a,b, Supporting Information). Consequently, the FE‐e‐skin possessed low strain interference (gauge factor < 0.03), maintaining consistent capacitance at 0% and 30% tensile strain (Figure 3d; Table S1, Supporting Information), even under dynamic stretching conditions during pressure sensing (Figure S15c, Supporting Information). Besides, owing to the excellent restorability of both the sensing unit and fibrous mat, the FE‐e‐skin exhibited outstanding cyclic stability, as demonstrated by the well‐preserved structural integrity in SEM images (Figure S16, Supporting Information) and the minimal sensitivity variation over 50000 loading cycles (Figure 3e; Figure S17, Supporting Information). Additionally, the FE‐e‐skin demonstrated a wide dynamic range (0–600 kPa) and enhanced pressure sensitivity (0.54 kPa^−1^), which can be attributed to the rough microstructure of the fiber‐entangled sensing units (Figure 3f; Figure S8b, Supporting Information).^[^ ^47^ ^]^ Further performance benchmarks included millisecond‐scale response (12 ms, Figure 3g) and low limit of detection (≈0.1 Pa, Figure S18, Supporting Information). The details regarding the characterization of sensing properties can be found in the Methods section. Moreover, the FE‐e‐skin exhibited excellent sensing stability (Figure S19, Supporting Information), characterized by a stable baseline and satisfactory pixel‐to‐pixel uniformity. Unlike the previously described performance metrics that were characterized using a single‐pixel FE‐e‐skin, the pixel‐to‐pixel uniformity was evaluated by randomly testing three pixels from a 100‐pixel array, as detailed in FigureS19c,d (Supporting Information). Overall, the fiber‐entangled architecture not only endowed the FE‐e‐skin with high pixel density and reduced strain interference, but also enhanced its pressure sensing performance.

In addition to its exceptional pressure sensing performance, biocompatibility is a critical attribute of wearable e‐skin.^[^ ^48^, ^49^, ^50^, ^51^, ^52^ ^]^ It can be rigorously assessed through permeability and cytotoxicity to mitigate adverse health effects, such as inflammation or allergies during prolonged wear.^[^ ^53^ ^]^ The FE‐e‐skin demonstrated high permeability, with air permeability (23.8 mm s^−1^) and moisture permeability (150.4 g m^−2^ day^−1^) comparable to commercial gauze and far superior to impermeable PDMS film commonly used in flexible electronics (Figure 3h; Figure S20 and S21 (Supporting Information) and Video S3 (Supplementary Video 3)). This biological parity is attributed to the high porosity of the fiber‐entangled architecture (Figure S22, Supporting Information). Furthermore, in vitro cytotoxicity studies were conducted using 4T1 cells as a model. Live/dead staining images (Figure S23, Supporting Information) and the CCK8 tests (Figure 3i; Figure S24, Supporting Information) demonstrated a remarkable increase in live cell numbers over time across all experimental groups, in stark contrast to 20% DMSO, which exhibited significant cytotoxicity. The combination of exceptional permeability and low cytotoxicity endowed the FE‐e‐skin with excellent biocompatibility, which was further confirmed by an on‐skin allergy test. Six samples (SEBS mat, sensitive layer, electrode layer, FE‐e‐skin, commercial gauze, and PDMS film) were attached to the forearms of one volunteer for two weeks. With the exception of PDMS, which caused obvious skin erythema, no adverse skin reactions were observed for the remaining samples, indicating the suitability of the FE‐e‐skin for prolonged wear (Figure 3j; Figure S25, Supporting Information). In addition, antibacterial experiments employing *Staphylococcus aureus* demonstrated the antibacterial property of the FE‐e‐skin, likely attributed to Ag nanoparticles within the electrode layer, further enhancing its wearing safety (Figure S26, Supporting Information).^[^ ^54^ ^]^ By combining strain‐insensitive pressure sensing with exceptional biocompatibility, the FE‐e‐skin establishes a reliable platform for high‐resolution wearable electronics tailored for tactile perception.

## 4 Strain‐Insensitive Pressure Imaging Based on the Fiber‐Entangled e‐skin

**Figure 4:**
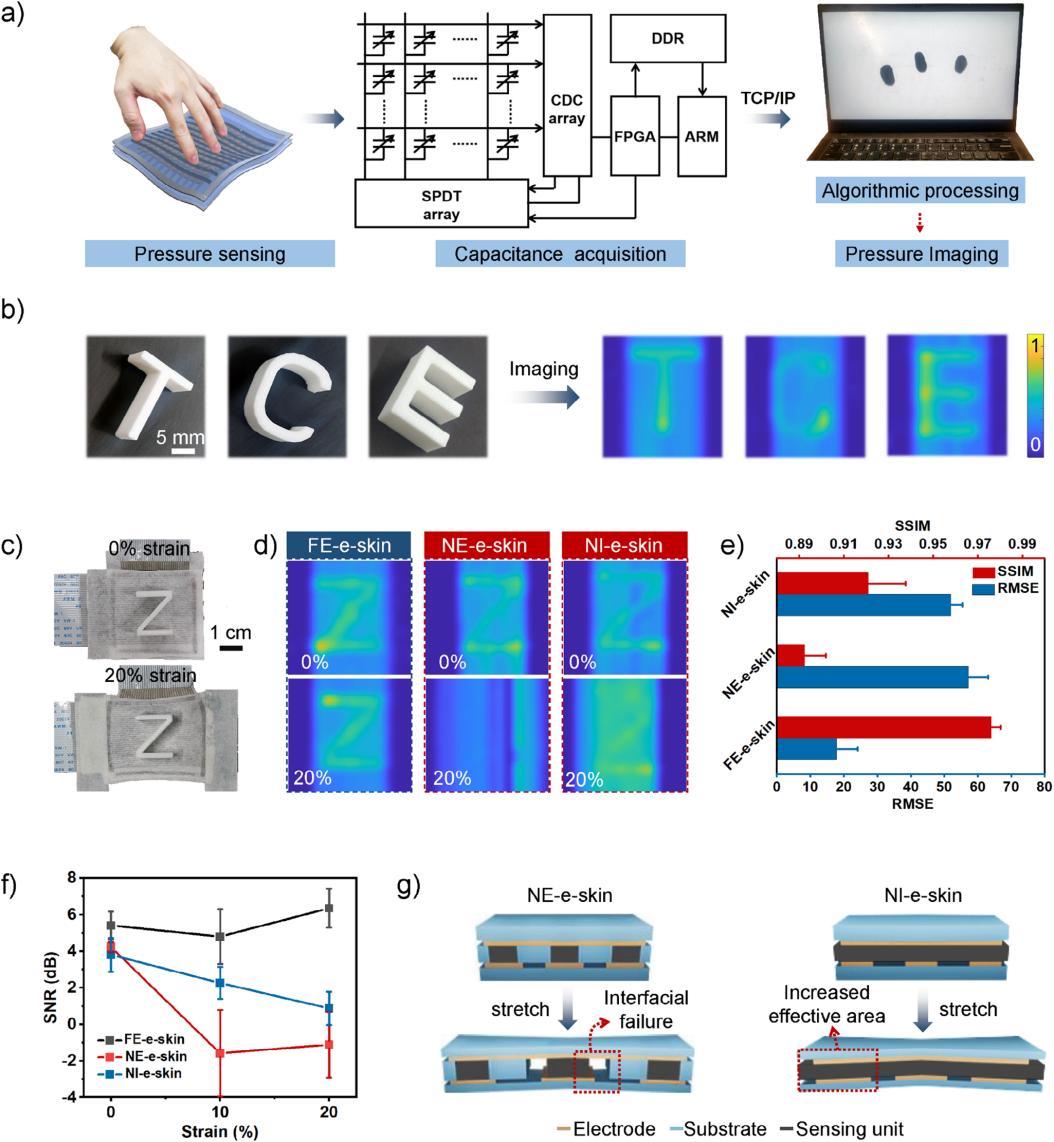
Strain‐insensitive pressure imaging based on the fiber‐entangled e‐skin. a) Schematic illustration showing the pressure imaging process of the FE‐e‐skin. b) The pressure image of three different letters collected by the FE‐e‐skin. c) Digital images showing the FE‐e‐skin capturing pressure images under 0% and 20% strain. d) The imaging results of the FE‐e‐skin, NE‐e‐skin, and NI‐e‐skin under 0% and 20% strain. e, f) Quantitative assessments of pressure images captured by different e‐skins under various stretching rates, using SSIM and RMSE (e), and SNR (f). g) Schematic illustration showing the negative impact of stretching on the NE‐e‐skin and NI‐e‐skin, indicating the significance of fiber‐entangled architecture in strain‐insensitive pressure imaging.

As a strain‐insensitive and high‐resolution pressure sensing device, the FE‐e‐skin was designed for wearable pressure imaging, which was demonstrated using a 1024‐pixel array with 1 mm spatial resolution. The arrayed capacitance signals were acquired using a custom high‐throughput acquisition system (Figure S27, Supporting Information). As schematized in **Figure** **4**a, the pressure imaging process consisted of three steps: i) pixel‐level capacitive pressure transduction, ii) high‐throughput capacitance acquisition via integrated circuit, and iii) algorithm‐driven data processing. This approach enables faithful reproduction of objects with millimeter‐scale feature structures (Figure 4b). Notably, during the imaging process, baseline drift occasionally occurred due to unreliable electrical connections between the FE‐e‐skin and external circuitry. When pressure was applied to the device, the contact resistance increased, leading to elevated capacitance measurements across all units in the affected column (Figure S28, Supporting Information). In this work, this issue was mitigated by manual baseline correction combined with a smoothing algorithm. However, employing more advanced electrical interconnection methods^[^ ^55^ ^]^ or implementing machine learning‐based data preprocessing could provide a better solution.

The pressure imaging reliability of the FE‐e‐skin was systematically evaluated by comparing its imaging results under 0% and 20% strain, with the latter fulfilling the requirements for wearable applications (Figure 4c). E‐skins featuring non‐entangled island arrays (NE‐e‐skin) and non‐island homogeneous sensing layers (NI‐e‐skin) were employed as references (see Methods section for fabrication details). It was found that the FE‐e‐skin consistently achieved high‐quality pressure imaging, accurately reproducing the shape of the applied object regardless of the stretching rate (Figure 4d). In contrast, the NE‐e‐skin suffered catastrophic failure under strain, while the NI‐e‐skins exhibited severe signal degradation, despite comparable imaging quality under 0% strain. Quantitative analysis using structural similarity index (SSIM) and root mean squared error (RMSE) underscored the advantages of the fiber‐entangled architecture in reliable pressure imaging (Figure 4e). The FE‐e‐skin demonstrated high SSIM and low RMSE under both 0% and 20% strain, outperforming the NE‐e‐skin and NI‐e‐skin. Moreover, the signal‐to‐noise ratio (SNR) of the FE‐e‐skin exhibited minimal correlation with the stretching rate, further highlighting the superiority of the fiber‐entangled architecture (Figure 4f). The high SNR was attributed to the signal crosstalk being limited to immediately adjacent points (Figure S29, Supporting Information). Beyond uniaxial stretching, the FE‐e‐skin also possessed strain‐insensitive pressure imaging performance under biaxial stretching (Figure S30, Supporting Information). In general, the fiber‐entangled architecture, which simultaneously provides low strain interference and high pixel density, enables strain‐insensitive pressure imaging essential for dynamic wearable tactile perception.

The mechanism underlying the enhanced imaging quality of the FE‐e‐skin, in comparison to the NE‐e‐skin and NI‐e‐skin, can be attributed to its fiber‐entangled architecture (Figure 4g). In the NE‐e‐skin, rigid islands without interfacial fiber entanglements experienced interfacial fracture under strain due to modulus mismatch (Figure S1, Supporting Information). This mismatch concentrated stress at island boundaries, propagating cracks that induced electrical short‐circuiting. Meanwhile, the NI‐e‐skin, featuring a homogeneous sensing layer, suffered baseline drift under strain as applied deformation altered the electrode‐sensing unit contact area, thereby reducing the SNR. In contrast, the FE‐e‐skin, owing to its fiber‐entangled architecture, mitigated stress concentration and deformation within the sensing units, thereby enabling strain‐insensitive pressure imaging. Integrating high biocompatibility and strain‐insensitive pressure imaging performance, the FE‐e‐skin holds great potential in wearable applications.

## 5 Proof‐of‐Concept Application using a Wearable Braille Point‐To‐Read System

**Figure 5:**
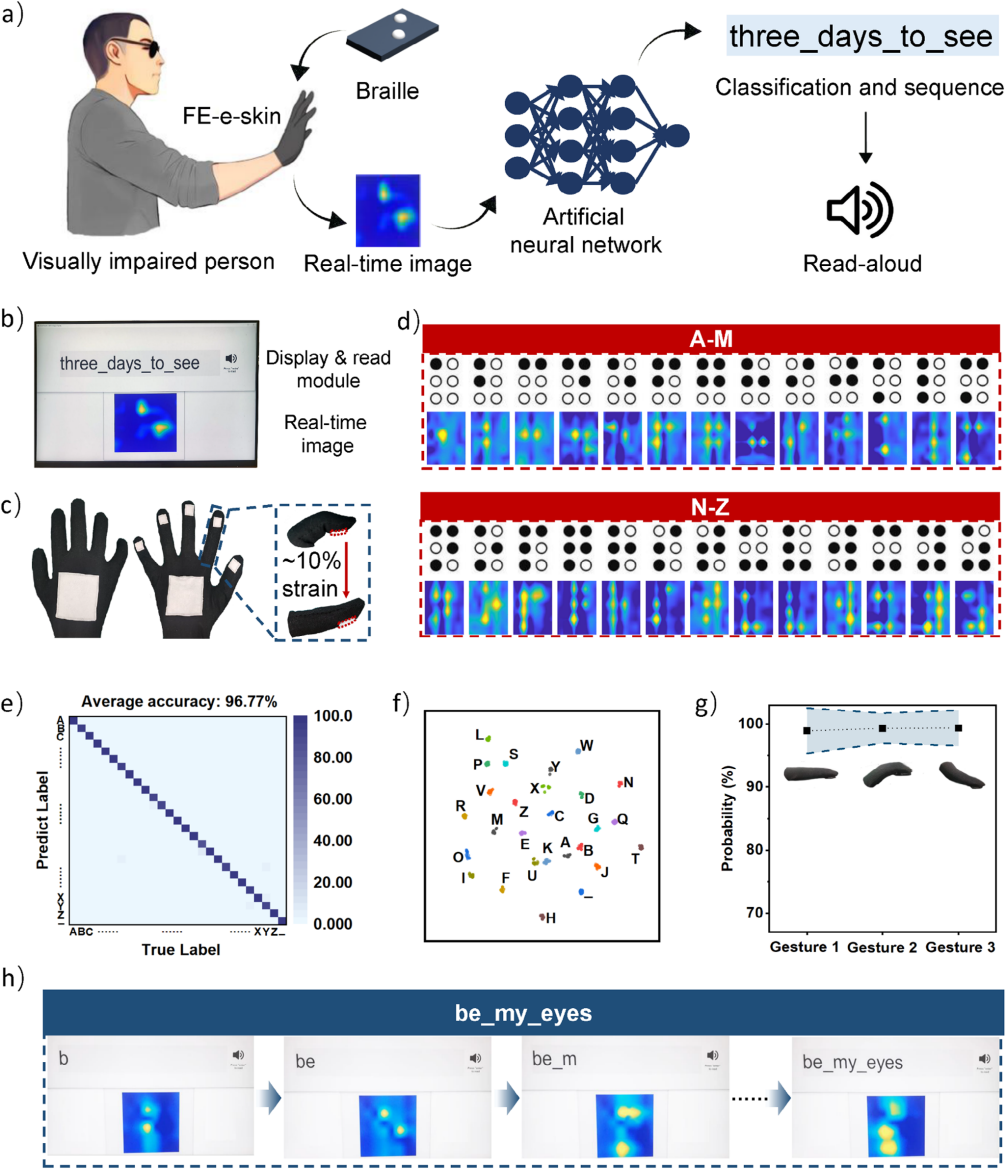
A proof‐of‐concept wearable Braille point‐to‐read system. a) Schematic illustration showing the Braille point‐to‐read process. b) An integrated user interface for the Braille point‐to‐read system. c) The tactile glove integrated with FE‐e‐skin for the Braille point‐to‐read system. d) The pressure images of various Braille characters from A to Z captured by the tactile glove. e) Confusion matrix showing the high classification accuracy for the Braille characters. f) T‐SNE plot from the Braille image dataset indicating the discriminative capability of the FE‐e‐skin. g) The classification accuracy for the Braille characters under different gestures. h) A typical point‐to‐read demonstration employing Braille phrases “be my eyes”.

To validate the performance of the fiber‐entangled architecture in practical applications, a 64‐unit FE‐e‐skin (1 mm spatial resolution) was integrated into a wearable glove (**Figure** **5**a,b; Figure S31, Supporting Information). By further combining the glove with a convolutional neural network, an artificial tactile perception system was achieved. Such an artificial tactile perception system successfully realized real‐time Braille character point‐to‐read, offering a transformative tool for visually impaired individuals to learn Braille. Reliable pressure sensing under dynamic hand movements was critical for this application, as gesture‐induced mechanical deformations during Braille interaction can cause strains of up to ≈10% in the e‐skin (Figure 5c),^[^ ^23^ ^]^ potentially leading to stretching crosstalk and diminishing imaging reliability. In this context, the strain‐insensitive pressure imaging performance of the FE‐e‐skin provided a distinct advantage over conventional e‐skins, enabling accurate imaging of all 26 Braille characters (A–Z) regardless of gestures (Figure 5d; Figure S32, Supporting Information).

Leveraging the strain‐insensitive imaging capabilities of the FE‐e‐skin, a Braille image dataset comprising over five hundred thousand high‐quality images was established, supporting the implemention of a ResNet‐34‐based transfer learning framework for Braille character classification. The dataset construction process was illustrated in Figure S33 (Supporting Information). Specifically, the raw data collected from the glove consisted of an 8×8 matrix, which subsequently underwent Gaussian smoothing and interpolation smoothing, yielding 16 000 high‐resolution patterns that that accurately represents the Braille characters. Then, the high‐resolution patterns were augmented by random translation, contrast adjustment, and Gaussian blur, ultimately forming the final dataset for model training and testing. Network parameters were optimized by minimizing cross‐entropy loss between predicted outputs and ground truth labels using the Adam optimizer. The exceptional imaging quality enabled by the fiber‐entangled architecture facilitated a classification accuracy exceeding 96% after five training epochs (Figure 5e). A comparison of the predicted and true labels for misclassified samples revealed that errors predominantly occurred among highly similar and complex Braille characters, such as Y (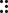), X (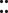), and Q (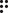) (see Figure S34 (Supporting Information) for details). Targeted data augmentation for these frequently confused classes, along with employing a deeper network, could potentially improve classification accuracy. Besides, projecting the high‐dimensional Braille images into 2D space via t‐distributed stochastic neighbor embedding (t‐SNE), it was observed that the recordings from different Braille characters naturally form distinctive clusters, demonstrating the discriminative capability of the FE‐e‐skin (Figure 5f). Systematic evaluation of the fiber‐entangled architecture's performance across varying finger configurations (extension, downward flexion, and upward flexion) demonstrated minimal correlation between gestures and predicted probability (Figure 5g).

The integration of the strain‐insensitive FE‐e‐skin with the high‐precision classifier enabled continuous Braille phrase recognition with maintained perceptual fidelity, despite dynamic hand positioning variations. To demonstrate this capability, two Braille phrases, namely “be my eyes” and “three days to see”, were successfully imaged and decoded with high accuracy (Figure 5h and Videos S4 and S5, Supplementary Video 4 and 5). These findings highlight the potential of the fiber‐entangled architecture proposed in this study for artificial tactile applications, particularly in wearable devices frequently subjected to stretching.

## 6 Conclusion

By emulating the architectural features of the elastin surrounding tactile receptors of human skin, we present a fiber‐entangled architecture capable of dissipating tensile stress during stretching, thereby resolving the modulus mismatch inherent in e‐skins featuring conventional island arrays. Stress dissipation arose from the strain‐induced reorientation of fibers from stochastic to ordered configurations. As a demonstration, FE‐e‐skins capable of highly reliable pressure imaging were successfully fabricated via stencil printing. Compared to other state‐of‐the‐art pressure‐sensing e‐skins, the FE‐e‐skin represented the only device strategy to date that simultaneously achieves high pixel density, low strain interference, and high permeability (Table S2, Supporting Information). The integration of the FE‐e‐skin with a high‐precision classifier enabled the development of a proof‐of‐concept wearable Braille point‐to‐read system, highlighting the potential of the fiber‐entangled architecture in artificial tactile applications. Considering that fabraication of the fiber‐entangled architecture was simple and scalable (Figure S35, Supporting Information), this strategy demonstrated commercialization potential, particularly when more reliable electrical connections^[^ ^55^ ^]^ were implemented to address baseline drift issues (Figure S28, Supporting Information). Overall, this fiber‐entangled architecture will emerge as an adaptable and robust platform for next‐generation humanoid sensing, offering exceptional reliability, high array density, and long‐term wearability.

## 7 Experimental Section

### Electrospinning of SEBS Mats

Commercial SEBS (Kraton G1726M, Kraton Corporation) was dissolved in tetrahydrofuran (THF) by magnetic stirring at 60 °C to produce a homogeneous viscous solution with a controlled SEBS‐to‐THF mass ratio of 1:5. The solution was loaded into a 10 mL syringe for electrospinning, conducted under ambient conditions with continuous fiber deposition onto a stainless steel collector. The applied voltage, feeding rate, and collecting distance were set to 10 kV, 10 mL h^−1^, and 15 cm, respectively. Upon carefully peeling the deposited fibers, the fibrous mat featuring reorientable fibers was obtained for further application.

### Stencil Printing of Fiber‐Entangled Arrays

EMIM TFSI (Sigma‐Aldrich) and thermoplastic polyurethane (TPU, BASF) were dissolved in N,N‐Dimethylformamide (DMF, Sigma‐Aldrich) by magnetic stirring (60 °C, 4 h) to fabricate a precursor solution for stencil printing. The mass ratio of EMIM TFSI to TPU was controlled at 3:5. For the printing of precursor solution on SEBS mats, various stencils with different island array patterns were prepared as masks. The SEBS mat was fixed on the printing table and covered by a stencil. Subsequently, the precursor solution was dropped onto the stencil and spread by a squeegee. The solution infiltrated the 3D fiber network of the mat and formed islands with fibers penetrating through them. After removing the stencil mask, a SEBS mat printed with a designed fiber‐entangled array was obtained. The printing accuracy was optimized by finely tuning the concentration of TPU from 15% to 35%.

### Stencil Printing of Stretchable Electrode Arrays

For stretchable pressure imaging applications, it is essential that electrode arrays possess both a low electrical conductivity and a low elastic modulus.^[^ ^56^, ^57^ ^]^ To achieve this, hierarchical structured Ag nanoparticles (AgNPs) and low‐modulus SEBS were employed as a conductive filler and a matrix material, respectively. The hierarchical structure of AgNPs reduced their percolation threshold within SEBS, allowing for conductivity at lower doping concentrations. The hierarchical structured AgNPs were fabricated using the following method. 5 g Silver trifluoroacetate (Nanjing Larui Innovation Technology Co., LTD, 99.9%) was evenly dissolved in 30 mL ethyl alcohol by magnetic stirring. Then, 7.5 mL hydrazine hydrate (Sinopharm Chemical Reagent Co., Ltd., 50% aqueous solution) was added dropwise to the ethanol to reduce the Ag^+^ and generate AgNPs. The AgNPs were collected by centrifugation, followed by washing the AgNPs three times with ethanol, using ultrasonication to ensure the complete removal of residual hydrazine hydrate.

To fabricate the precursor solution for stencil printing, the AgNPs and SEBS were dispersed in cyclopentyl methyl ether by magnetic stirring at room temperature for 2 h. The mass ratio of AgNPs to SEBS was controlled at 4:1. Various stencils with different parallel line patterns were prepared as masks. The SEBS mat was fixed on the printing table and covered by a stencil. The precursor solution was dropped onto the stencil and spread by a squeegee. After removing the stencil mask, a SEBS mat printed with designed stretchable electrode arrays was obtained. The printing accuracy was optimized by finely tuning the concentration of SEBS.

### Fabrication of e‐skins

For the fabrication of the FE‐e‐skin, two stretchable electrode arrays and a fiber‐entangled array were aligned and bonded using pre‐cut stretchable adhesive tape (30 µm thick). Both the electrode line pitch and the fiber array pixel pitch were set to 1 mm, matching the pixel density of human fingertip skin (Figure 3b). Commercial flexible flat cables (FFCs) with a 1 mm pitch were bonded to the stretchable electrode arrays in a one‐to‐one wire correspondence using anisotropic conductive film, enabling robust electrical interfacing between the e‐skin and external circuitry (Figure S24, Supporting Information).

The NE‐e‐skin and NI‐e‐skin reference devices followed the same fabrication process as the FE‐e‐skin, with variations only in the sensitive layer. For the NE‐e‐skin, a unidirectionally aligned SEBS mat served as the substrate for the sensitive layer, produced through electrospinning on a drum collector. This pre‐aligned architecture eliminated fiber entanglement within the sensitive layer. In addition, the NI‐e‐skin incorporated a continuous EMIM TFSI/TPU film fabricated by casting as the sensitive layer.

### Characterization of Strain‐Insensitive Pressure Sensing Property

The morphologies of the samples were investigated by a scanning electron microscope (SEM, Quanta 200, Hitachi) and an optical microscope. The chemical composition of the samples was investigated by X‐ray photoelectron spectroscopy (EscaLab 250Xi, Thermo) and a Fourier Transform Infrared Spectrometer (Spectrum TWO, PerkinElmer). The mechanical properties of the samples were tested using an Instron E1000 universal testing system, in which samples were cut into strips with dimensions of 60 × 5 mm for the test. The electrical resistances of the samples under different stretching states were investigated by a Keithley 2400 Sourcemeter. The capacitance‐to‐pressure response of the e‐skins was tested by a customized LabVIEW program connected to the LCR meter (E4980a, Keysight). The applied pressure was controlled and measured by the Instron E1000 universal testing system. Apart from the response time test, all the capacitance responses were measured at a frequency of 1 kHz and oscillator voltage level of 1 V without dc bias.

The stretching crosstalks were measured by Gauge factor using equation:^[^ ^58^ ^]^

(1) $$GF=\frac{\Delta S/S_{0}}{\varepsilon }$$

where ∆S was the electrical signal variation under tensile strain, *S*_0_ represented the initial signal, and ε was the tensile strain. Depending on the device type, *S* corresponds to current (I), voltage (V), or resistance (R).

To characterize strain interference during pressure sensing (Figure 3d,e), graded pressures ranging from 1 to 50 kPa were applied to a FE‐e‐skin under both 0% and 30% tensile strain. During testing, the stretched e‐skin was fixed at both ends with adhesive tape on a universal testing machine to ensure a constant strain throughout the measurement. Besides, the dynamic range and pressure sensitivity was evaluated by measuring the pressure‐capacitance response curve of the FE‐e‐skin over a broad pressure range (Figure 3f), where the span of the pressure axis defines the dynamic range, and the slope of the curve represents the sensitivity. In addition, the response time was measured by quickly loading a 50 g weight on the sensor. During this process, the LCR meter was operated at a frequency of 1 MHz with its data correction and display functions disabled to achieve the minimum sampling interval. Furthermore, to characterize limit of detection, paper sheets of varying thicknesses were employed to apply small pressures.

### Pemeability Test and Porosity Calculation

The air permeability value of all the samples was tested according to GB/T 1038–2000 using an air permeability tester (Labthink International, Inc., BTY‐B2P). The output airflow rate (mm s^−1^) represents the air permeability of samples. The moisture transmission rate (g m^−2^ day^−1^) was determined by measuring the weight loss of the water vapour in the beaker with its opening firmly covered by the tested specimen (testing duration of 72 h). Commercial medical gauze and PDMS films were employed as controls for comparison.

The porosity of the SEBS mat was calculated based on Archimedes’ principle using equation:

(2) $$P_{SEBS\;}=\left(1-\frac{V_{true}}{V_{apparent}}\right)\times 100\%$$

where *V_apparent_* was the apparent volume and *V_true_* was the true volume of the tested mat. The *V_apparent_* was calculated based on the dimension of the sample. The *V_true_* was calculated by dividing the mass of the SEBS mat by its true density. The porosities of the fiber‐entangled array and the stretchable electrode array were calculated based on the ratio of the permeable area to the total area, taking *P*_SEBS mat_ as the reference.

### Cell Cytotoxicity and Skin Irritation Tests

4T1 mouse mammary cancer cells were used to evaluate the in vitro cytotoxicity of the samples. The tested specimens were cut into 0.25 cm^2^ and added to the wells containing the prepared cell suspension. Fresh complete medium, commercial medical gauze (0.25 cm^2^), and 20% DMSO were chosen as the control groups. The samples were cultured at 37 °C with 5% CO_2_ for different time periods (24, 48, and 72 h). Cytotoxicity was assessed using live/dead staining and CCK‐8 assays. For the live/dead assay, 200 µL of a staining solution containing 2 µM calcein‐AM and 8 µM PI in PBS was added and incubated for 15–20 min at 4 °C in the dark. After rinsing with PBS, fluorescence imaging was performed using a Leica DMI8 fluorescent microscope (Leica, Germany). For the CCK‐8 assay, 500 µL of CCK‐8 solution (MCE, USA) was added to each well and incubated for 2 h at 37 °C. Absorbance at 450 nm was measured using a microplate reader (EPOCH2, BioTek, USA).

The skin irritation test of the samples was carried out on a volunteer's skin. Six samples (the SEBS mat, fiber‐entangled array, stretchable electrode array, FE‐e‐skin, commercial medical gauze, and non‐permeable PDMS film) were attached to the skin of the forearms of the volunteer, and the edges were secured with acrylic adhesive (Walker Tape Co., Ltd). After being worn for a duration of two weeks, the covering materials were removed from the skin. The skin was photographed before and after the test so that the appearance could be compared.

### Strain‐Insensitive Pressure Imaging

To achieve pressure imaging, high‐throughput acquisition of arrayed capacitance signals and real‐time signal processing were essential. Consequently, a custom‐designed signal acquisition circuitry and a dedicated signal processing Matlab program were developed. The circuitry was mainly composed of a single pole double throw (SPDT) array, a capacitance‐to‐digital converter (CDC) array, a heterogeneous computing platform (ZYNQ‐7000, which includes FPGA and ARM), and a DDR storage unit (Figure 4a). Control signals generated by the FPGA sequentially scanned matrix columns via the SPDT array, afterwhich signals from all units of the selected column were digitized by the CDC. The unselected columns were connected to the SHLD pins of the CDC via the SPDT to minimize crosstalk between the selected column and unselected columns. Upon completing the scan of all columns, the data was processed and transmitted to Matlab for display. The characters used for applying pressure were fabricated via 3D printing using Poly Lactic Acid (PLA). It should be noted that during signal acquisition in a 32×32 array, the performance constraints of the CDC chip (FDC1004) may induce column‐specific baseline capacitance deviations. Nonetheless, these systematic errors can be mitigated by employing machine learning‐assisted image enhancement techniques (Figure S25, Supporting Information). Here, in order to accurately reflect the advantages of the FE‐e‐skin over traditional e‐skins, baseline offsets were manually calibrated following the principle of preserving pattern characteristics. The strain interference of pressure imaging was evaluated by structural similarity index (SSIM), root mean square error (RMSE), and signal‐to‐noise ratio (SNR) of images under different stretching rates. All parameters were directly computed using MATLAB, ensuring algorithm consistency across datasets.

### Assembly of the Tactile Glove

A commercially available elastic glove was employed to construct the tactile glove. The 8×8 FE‐e‐skin with a spatial resolution of 1 mm was attached to the fingertip of the glove using stretchable tape. Two 1‐cm incisions were made near the glove's fingertip to facilitate the routing of the flexible flat cable (FFC) that interfaces the e‐skin with external circuitry (Figure S28, Supporting Information). The FFC was laid along the interior surface of the glove. This strategic placement was designed to eliminate motion‐induced signal artifacts, ensuring reliable signal transmission, while also maintaining the mechanical compliance of the glove during dynamic manipulation tasks.

### Model Training for Braille Point‐To‐Read

The dataset comprise over 500 000 images were divided into training, validation, and testing sets in an 8:1:1 ratio. The Braille characters used for image acquisition were fabricated via 3D printing using PLA, with a dot spacing of 2.5 mm, dot height of 0.8 mm, and dot diameter of 1.6 mm. For the model training, transfer learning was utilized using the pre‐trained ResNet‐34 model (accessible at https://download.pytorch.org/models/resnet34‐333f7ec4.pth), which was tailored to a new task by replacing the fully connected layer. The t‐SNE visualization was performed by extracting high‐dimensional features from the model's final layer and reducing these features to 2D. Both the t‐SNE and confusion matrix were derived using data from the testing set.

### Statistical Analysis

All plotting and analysis were performed using Origin 2021 software. All characterizations were conducted with a sample size of at least three. For biocompatibility and imaging analyses, data are presented as mean ± standard deviation (SD). The data for cyclic stretching and pressure testing were pre‐processed to identify outliers caused by instrumental fluctuations. Extreme values were visually inspected and considered out of a reasonable range. No data were excluded unless there was clear evidence of measurement error.

## Conflict of Interest

The authors declare no conflict of interest.

## Author Contributions

R.Q. and Z.M. conceived and designed the experiments. R. Q. performed the experiments. M.J. and M.Z. developed the circuit boards. N. Z. performed the materials characterization. R.Z. and H.H.trained the models. Y. Z., F. C., M. C., and J. J. analyzed the data. H.J. performed the cell experiments. R. Q. worte the paper. Z. M. supervised the whole project. All authors discussed the results and commented on the manuscript.

## Supporting information



Supporting Information

Supplementary Video 1

Supplementary Video 2

Supplementary Video 3

Supplementary Video 4

Supplementary Video 5

## Data Availability

The data that support the findings of this study are available from the corresponding author upon reasonable request.
