# Supplementary material for: A Biomimetic Fiber‐Entangled Permeable Electronic Skin for Strain‐Insensitive and High‐Resolution Tactile Sensing
Source: Adv Sci (Weinh). 2025 Aug 28;12(43):e12111. doi: 10.1002/advs.202512111 (PMC12631820; doi:10.1002/advs.202512111)
Supplement: Supplementary file 1 — Supporting Information [file ADVS-12-e12111-s005.docx]

Supporting Information

**A biomimetic fiber-entangled permeable electronic skin for strain-insensitive and high-resolution tactile sensing**

Ruixiang Qu^1^, Menghui Ji^2^, Ningjing Zhou^1,3^, Rongdi Zhang^4^, Huijiao Ji^5^, Min Zou^2^*, Huacheng He^4^, Yu Zhang^1^, Fuguang Chen^1,3^, Mengjia Chen^1,3^, Jiujiang Ji^6^, Zhijun Ma^1,3^*

1 Research Center for New Materials Computing, Zhejiang Lab, Hangzhou, China

2 Research Center for Computing Sensing, Zhejiang Lab, Hangzhou, China

3 School of Materials Science and Engineering, Zhejiang University, Hangzhou, China

4 Oujiang Laboratory, Wenzhou, China.

5 Department of Pharmacology, School of Pharmacy, Southwest Medical University, Luzhou, China.

6 Department of Chemistry, Tsinghua University, Beijing, China

E-mail addresses: zoum@zhejianglab.org, zhijma@zhejianglab.com


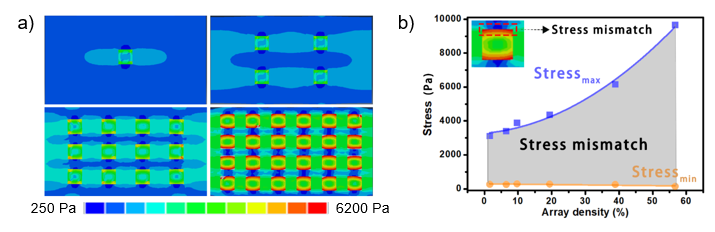


**Figure S1. Finite-element simulation of traditional island arrays with nonporous substrate. a)** Stress distribution in arrays with varying island densities under 30% strain. **b)** Stress mismatch escalated significantly as island density increased. The elastic modulus of the stretchable substrate and rigid island were 150 kPa and 1 GPa, respectively, with Poisson’s ratios of 0.3 for both materials.

**Figure S2. Micrograph depicting the dynamic stretching process of a fiber-entangled array.** Strain localizes predominantly in the low-modulus substrate due to the significant modulus mismatch between the islands and substrate, while the high-modulus islands exhibited negligible strain.


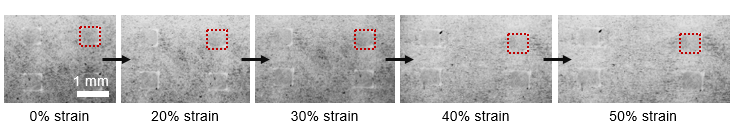


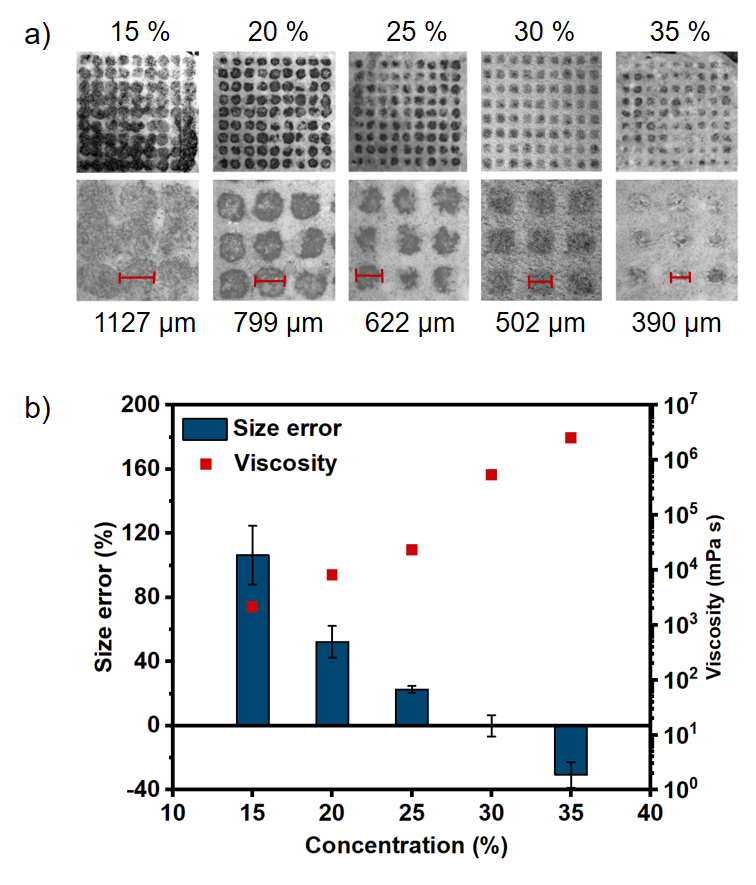


**Figure S3. Spatial uniformity control in stencil-printed islands via precursor viscosity optimization.** **a)** Micrographs of arrays fabricated from precursor solutions with varying TPU concentrations (solvent: DMF) reveal pronounced differences in spatial uniformity. **b)** Relationship between TPU concentration, solution viscosity, and printing resolution, identifying 30% TPU as the optimal concentration.


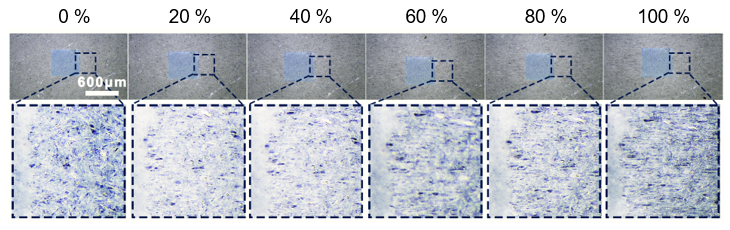


**Figure S4.** Optical micrographs revealing fiber orientation changes at the island-substrate interface under varying stretching rates.


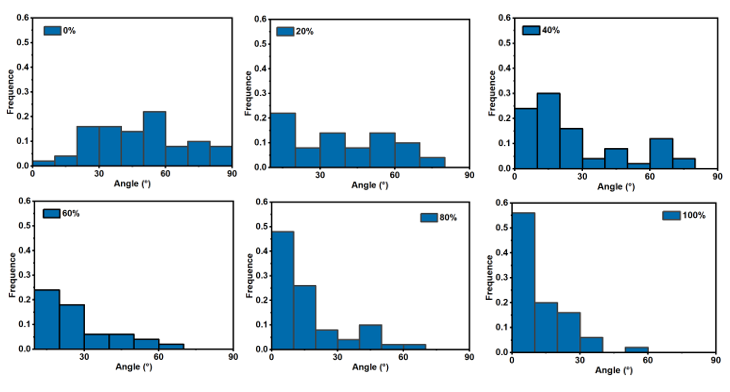


**Figure S5. Quantitative analysis of fiber orientation distribution upon stretching.** The fiber orientation was quantified by the angular deviation of the fibers relative to the strain direction. As strain increased from 0% to 100%, fibers transitioned progressively from random orientations to alignment parallel to the strain axis.

**Figure S6:** **The effect of fiber parameters on mitigating stress concentration**. a)-c) the SEM images of fiber-entangled sensing arrays with different fiber diameters and initial entanglement densities before stretching. d)-f) the SEM images of fiber-entangled sensing arrays after cyclic stretching. g)-i) The sensing arrays withstood more than 1000 stretching cycles under 100% strain regardless of fiber diameter and initial entanglement density variation.


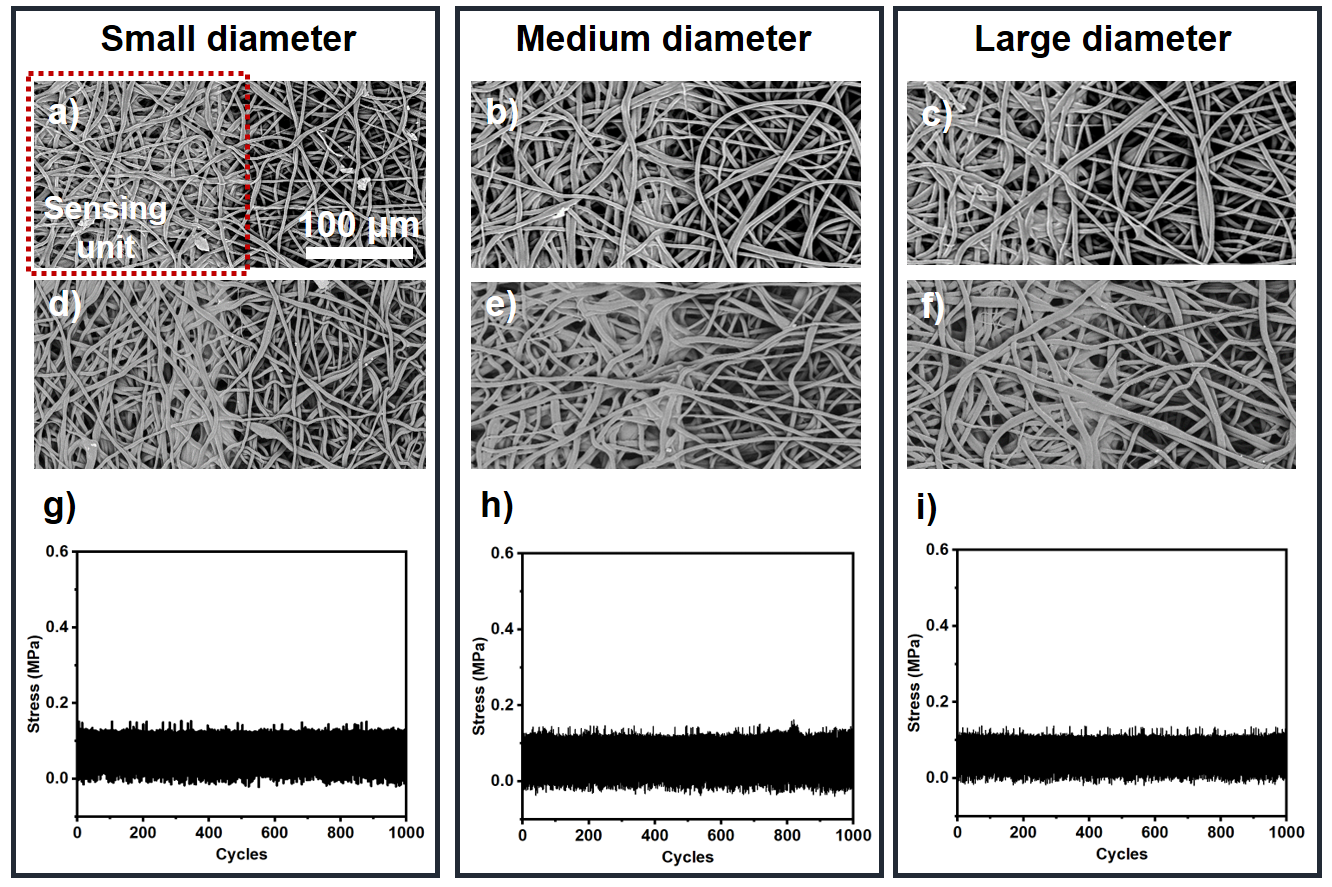


**Additional discussion of Figure S6:**

The effects of fiber parameters on stress concentration mitigation was observed. By varying the solution feed rate (4 ml h^-1^, 7 ml h^-1^, and 10 ml h^-1^) during electrospinning, three SEBS mats with varying fiber diameters were obtained. As fiber diameters increased, a corresponding change in fiber density and elastic modulus was observed.

Based on the mats, three sensing array samples with varying initial entanglement densities were subsequently fabricated. The ability to mitigate stress concentration was evaluated through cyclic tensile tests (100% strain) and SEM images taken before and after 1,000 stretching cycles. Prior to the cyclic tests, 10 pre-stretching cycles were applied to the samples to achieve mechanical stabilization. As shown in Figure S6, all arrays exhibited effective stress concentration mitigation, with their morphologies and mechanical properties remaining stable after cycling tests.

As a result, fiber parameters exhibit low correlation with stress concentration mitigation capability.


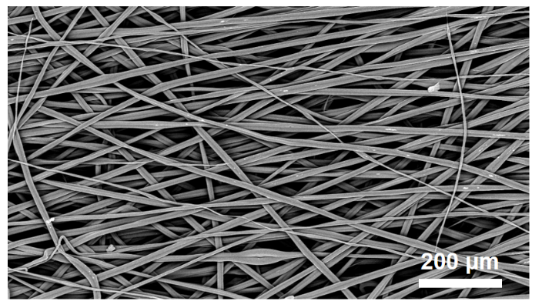


**Figure S7:** The SEM image of the fibrous mat with quasi-unidirectional fiber alignment fabricated through drum-assisted electrospinning.


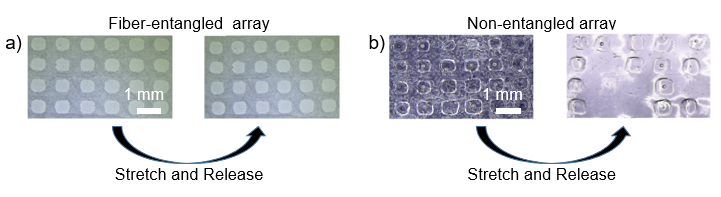


**Figure S8. Comparison of a single stretching cycle for fiber-entangled and non-fibrous arrays.** The fiber-entangled array exhibited structural integrity after one stretching cycle, whereas the non-fibrous array showed detachment of islands. This was because the fiber-entangled array could effectively dissipate stress through fiber reorientation, a mechanism absent in non-fibrous arrays. This highlighted the enhanced mechanical stability and superior performance of fiber-entangled arrays in e-skin applications..


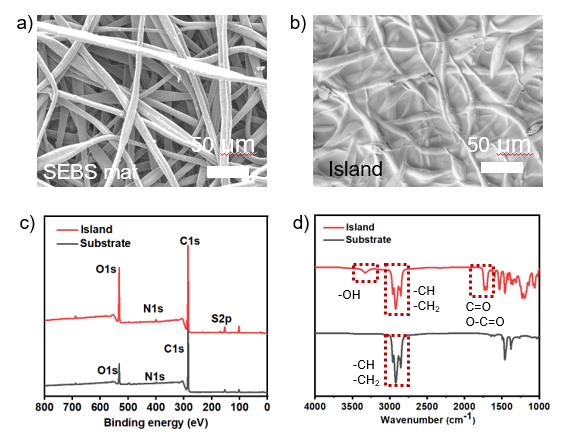


**Figure S9. Morphology and composition characterization of the fiber-entangled array composed of TPU/EMIM TFSI island and SEBS mat substrate. a)** SEM image showing the morphology of the SEBS mat, characterized by randomly oriented fibers. **b)** SEM image showing the morphology of the TPU/EMIM TFSI island. TPU/EMIM TFSI uniformly covered the fiber of the SEBS mat, forming a rough architecture. **c)** X-ray photoelectron spectra of the substrate and islands. **d)** Fourier Transform infrared spectra of the substrate and islands.

**Figure S10. Stretchability of the FE-e-skin. a)** Stress-strain curve of the FE-e-skin. The e-skin possessed a low elastic modulus (~617 kPa) and a high fracture elongation (> 900%). **b)** The FE-e-skin withstood more than 3000 stretching cycles under 100% strain. The exceptional mechanical properties ensure reliable performance in wearable applications requiring repeated dynamic deformation.


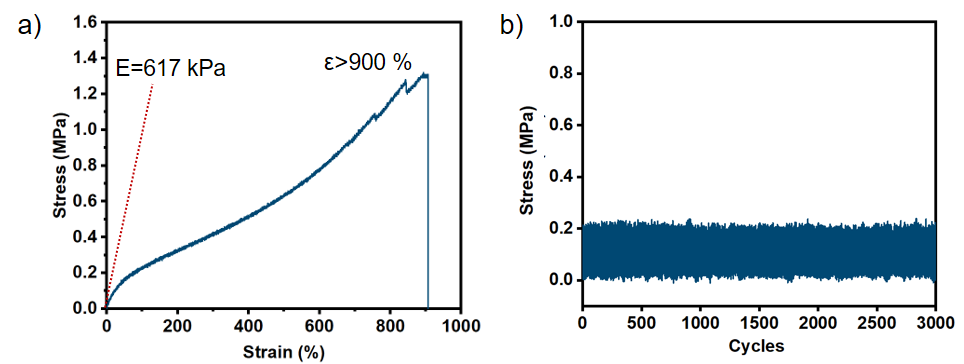


**Figure S11:** Both the sensing array and the electrodes exhibited slight pitch variation after cyclic stretching. However, since the pitch variations in the sensing array and the electrode layer were identical, the electrodes and sensing islands remained precise alignment. This was because the pitch variations were primarily caused by SEBS viscoelastic relaxation during the initial stretching cycles, and the length of the SEBS layer between the sensing array and electrodes was uniform.


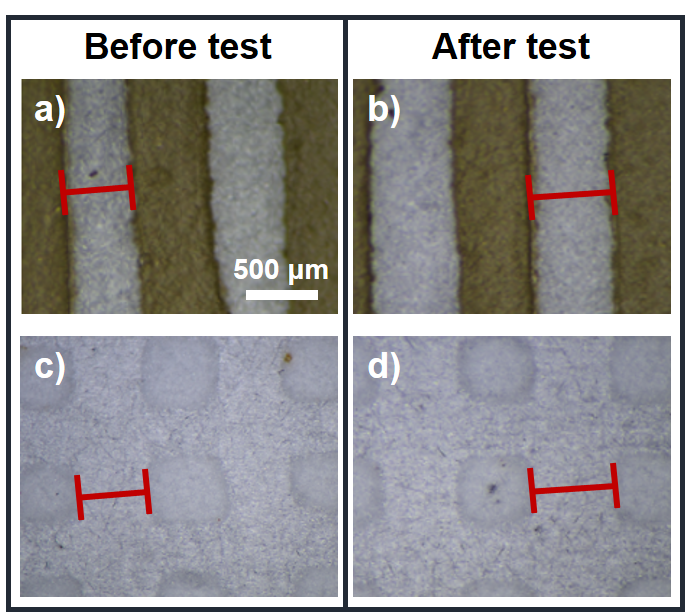

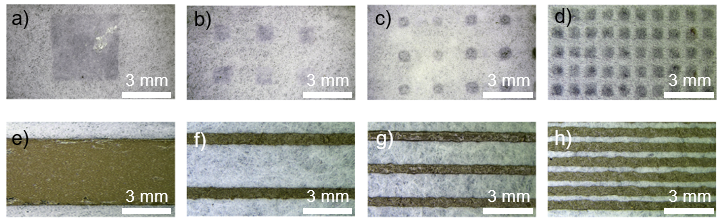


**Figure S12. Programmable pixel density of the FE-e-skin. a) -d)** Fiber-entangled island with tunable sizes and pitches. **e)-f)** Stretchable electrode arrays with various line widths and pitches. The pixel density can be regulated by adjusting the stencil patterns, enabling versatile functionality in the FE-e-skin.

**Figure S13. The electro-mechanical properties of the electrode layer a)** Stress-strain curve of the electrode layer. The electrode layer possessed a low elastic modulus (~560 kPa) and a large fracture elongation (> 900%). **b)-c)** The SEM images of the Ag/SEBS before and after 3000 stretching cycles. **d)-e)** The SEM images of the commercial stretchable silver paste (SHAREX, AS7126) before and after 1000 stretching cycles. **f)** The embedded structure between the Ag/SEBS and SEBS mat. **g)** Resistance-strain curve of the electrode layer. The electrode layer possessed low resistance under stretching (<15 Ω at 30 % strain).


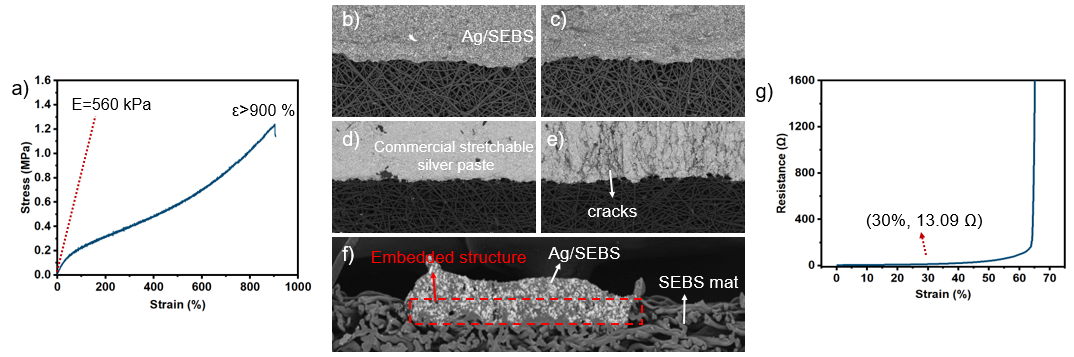


**Figure S14. Electric double-layer capacitive response of the FE-e-skin. a)** Schematic illustration of the charge transfer behavior related to the electrical double layer during pressure sensing. **b)** Equivalent circuit of a single sensing unit. **c)** UAC in a single sensitive unit as a function of test voltage at varying frequencies. **b)** UAC in a single sensitive unit as a function of frequency under a fixed test voltage (1V).


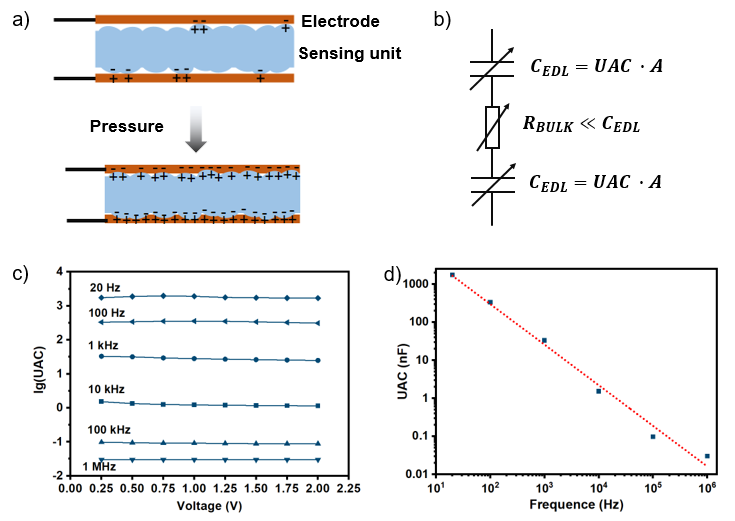


**Additional discussion of Figure S14:**

The FE-e-skin is an iontronic pressure sensor whose sensing mechanism is associated with the electrical double layer (EDL). Specifically, when the electrode comes into contact with the sensing unit, ions within the sensing unit are attracted to the electrode, forming a region known as the EDL at the interface between the sensing unit and the electrode (Figure S14a). The EDL can be modeled as a conventional parallel-plate capacitor (Figure S14b), where both the dielectric constant and the separation distance between the plates are solely determined by the material properties. Consequently, the capacitance depends only on the contact area between the electrode and the sensing unit. As pressure increases, the contact area expands, leading to an increase in capacitance.

This sensing principle was supported by the frequency-dependent unit-area capacitance (UAC) behavior of the sensitive unit, which aligned with the characteristic dynamics of electric double-layer capacitance (Figure S14c). Moreover, the remarkably high UAC of ~32 nF (measured at 1 V and 1 kHz) ensured that sensing elements remained measurable even at sub-millimeter sizes, facilitating the fabrication of high-density pressure sensor arrays.

**Figure S15. The pronounced modulus disparity suppressed strain interference. a)** Stress-strain curve of the SEBS mat substrate. The SEBS mat possessed a low elastic modulus (~110 kPa). **b)** Stress-strain curve of the sensitive unit. The sensitive unit possessed a high elastic modulus (~6.7 MPa). **c)** The low strain interference under dynamic tensile strain. The strain interference under dynamic condition was characterized by applying pressure to the device while simultaneously imposing dynamic strain by a compact tensile stage. Dynamic strains of 30% and 50% resulted in capacitance variations of approximately 0.27% and 2.19%, respectively.


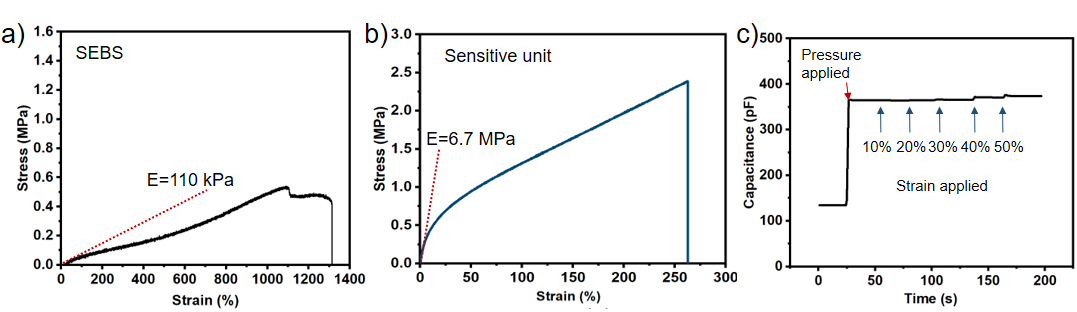

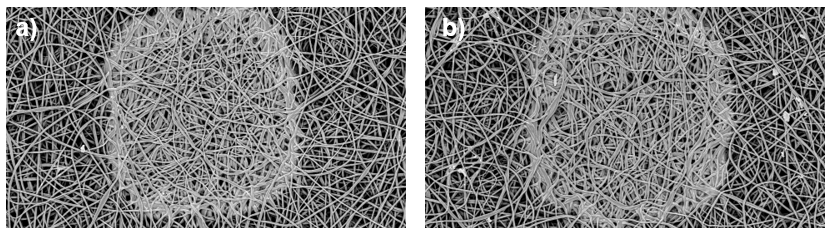


**Figure S16:** The SEM images of the sensing unit before and after 50000 compression cycles.

**Figure S17:** **The capacitance values from the first 1000 cycles and last 1000 cycles of cyclic pressure testing.** There was a measurable baseline drift. However, this baseline drift did not influence the measured pressure. This insensitivity occurs because the pressure signal was directly determined by ΔC/C. In iontronic sensors, ΔC/C depends solely on changes in the contact area between the electrode and the dielectric layer, independent of the initial capacitance value.


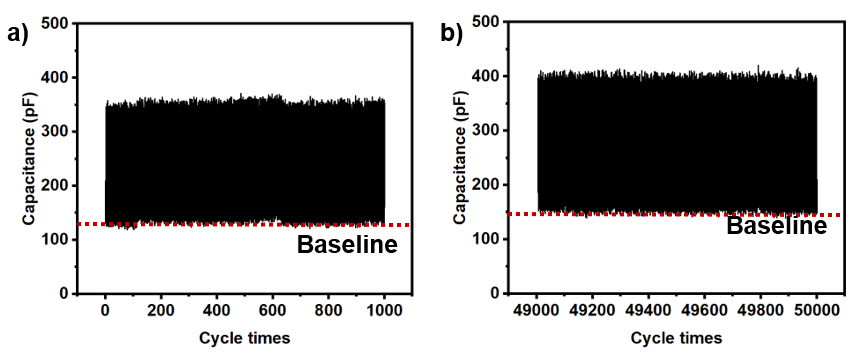

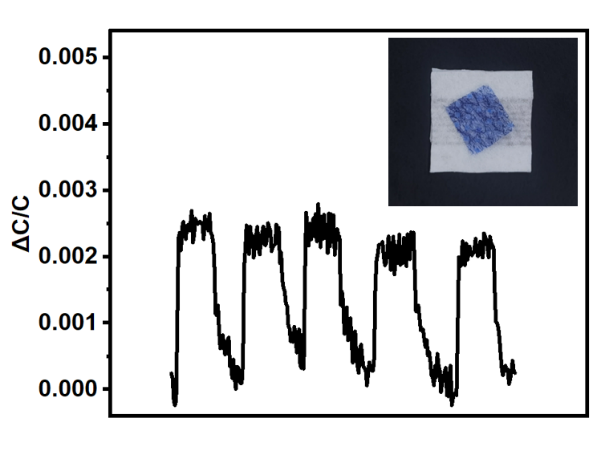


**Figure S18. Limit of detection of the FE-e-skin.** The FE-e-skin was capable of detecting pressure as low as 0.1 Pa, generated using paper with a mass of 10 g m⁻² (as shown by the inset photograph). This low limit of detection originated from the rough surface morphology of the fiber-entangled islands.

**Figure S19. Sensing stability of the FE-e-skin. a)** The FE-e-skin retained stable baseline during proximity (2 mm distance) to objects of diverse material compositions. **b)** The FE-e-skin exhibited stable baseline over time. c) The variation in sensitivity among pixels was within an acceptable range. d) The pixel-to-pixel variation in response time was confined to the millisecond range.


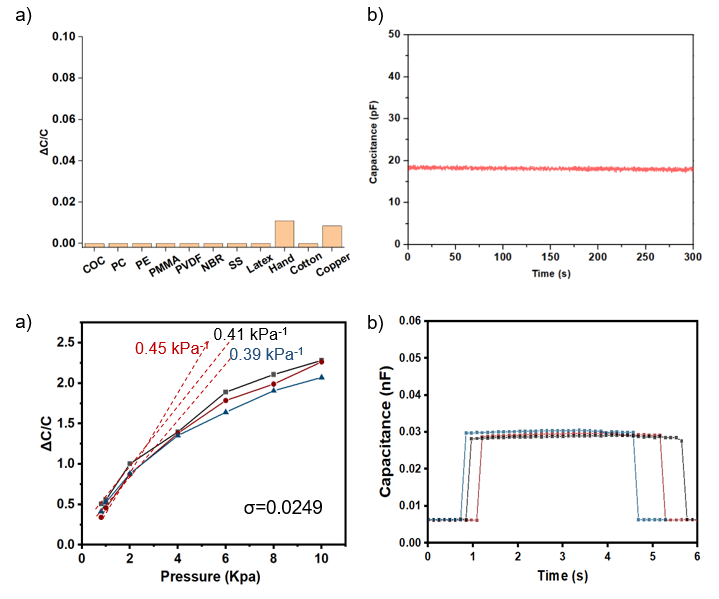


**Additional discussion of Figure S19:**

To assess the pixel-to-pixel uniformity, we fabricated a 100-pixel array (100 unit cm^-2^) and randomly selected three pixels to characterize their sensing properties. Pressure was applied locally to individual pixels using a 3D-printed resin probe with a tip diameter of 1 mm.

The sensitivity was obtained by measuring the capacitance change of the sensing units in the low-pressure range (1-10 kPa). As shown in Figure 19c, within the same device, the variation in sensitivity among pixels was within an acceptable range. For the device used in this test, the maximum sensitivity exhibited a standard deviation of 0.0249. In addition, the response time was measured at a frequency of 1 MHz, at which the LCR meter can reach its maximum sampling frequency. As shown in Figure 19d, the response times of all tested units reached the detection limit of the LCR meter (12ms). This suggests that the pixel-to-pixel variation in response time was confined to the millisecond range, which was acceptable for practical applications.


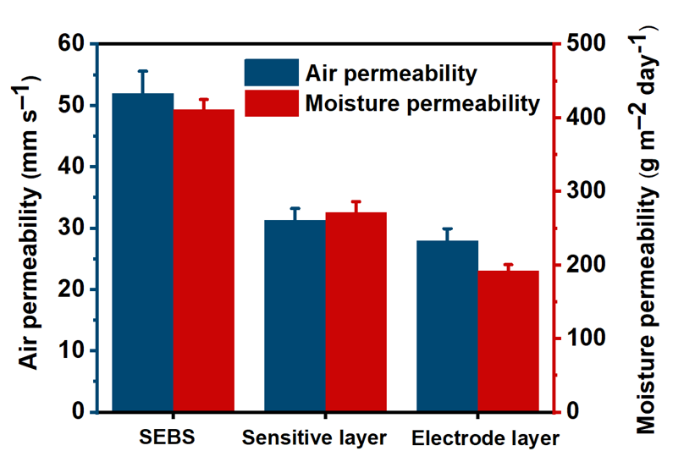


**Figure S20**. **Air and moisture permeability of FE-e-skin component layers.** The SEBS mat substrate, fiber-entangled sensitive layer, and electrode layer possessed high permeability.

**Figure S21**. **Photographs showing the moisture permeability of the FE-e-skin.** Five different samples were sealed in the mouth of a 3 cm-diameter breaker. The flask was filled with hot water and placed on hot plate to continue heating. Clean glass slides positioned ~10 mm above the samples revealed condensation in all cases except for the PDMS control, indicating the outstanding moisture permeability of the FE-e-skin.


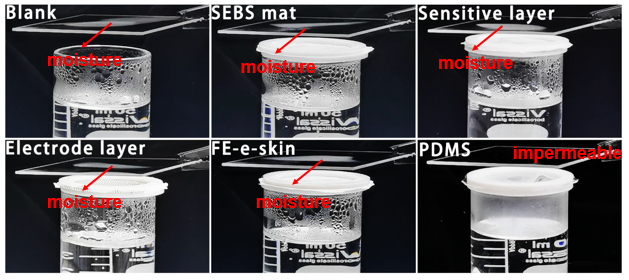


**Figure S22**. **Porosity of FE-e-skin component layers.** The porosity was calculated based on Archimedes’ principle (as described in the Methods section). The high porosity of each individual layer contributes to the FE-e-skin's enhanced permeability.


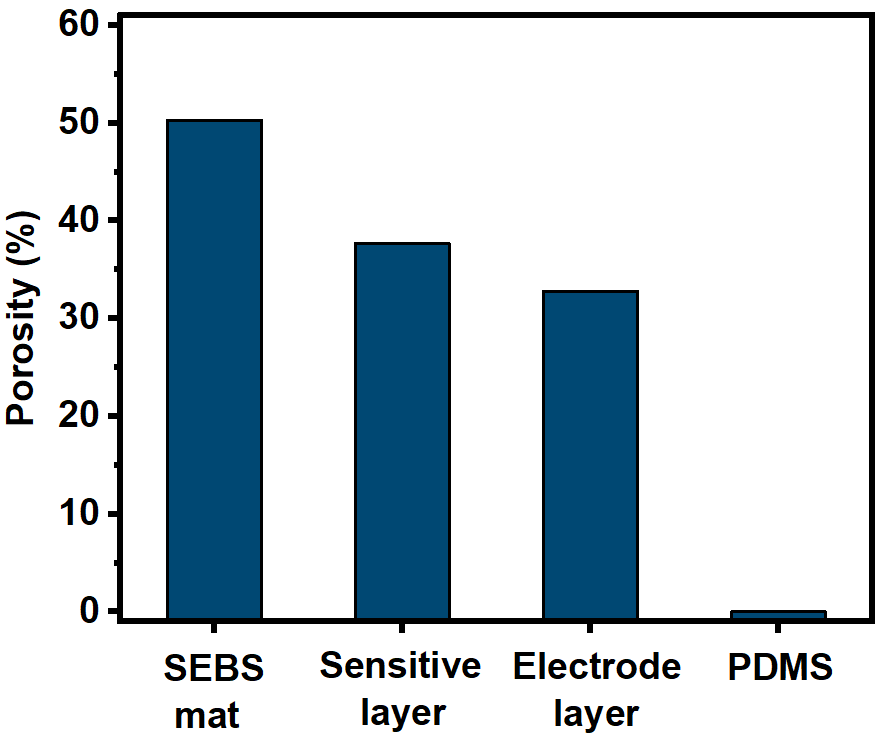

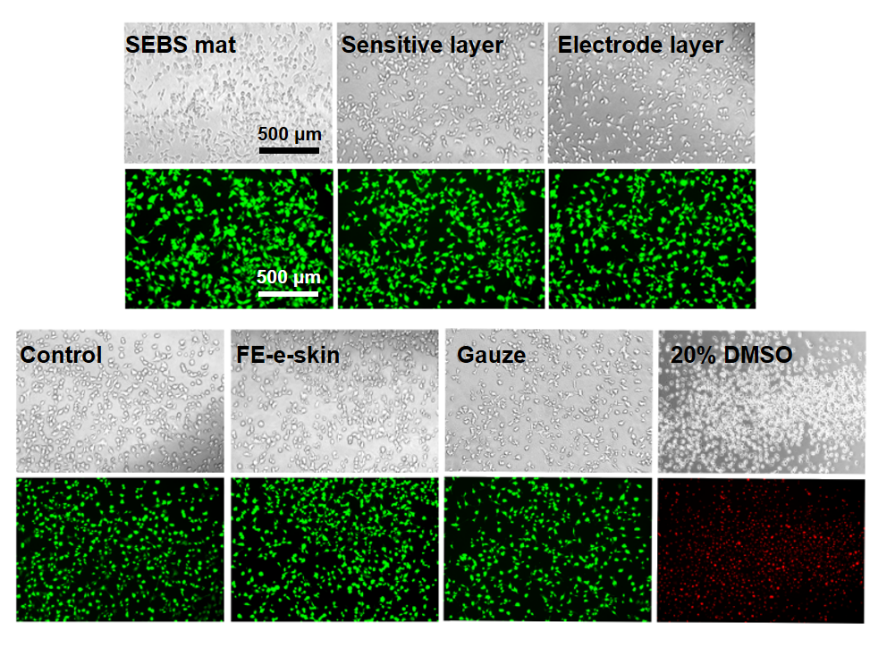


**Figure S23**. Bright-field and fluorescent images of cells cultured in the incubation medium with the control sample, SEBS mat, sensitive layer, electrode layer, FE-e-skin, gauze, and 20% DMSO. The FE-e-skin possessed low cytotoxicity that was suitable for wearable applications.


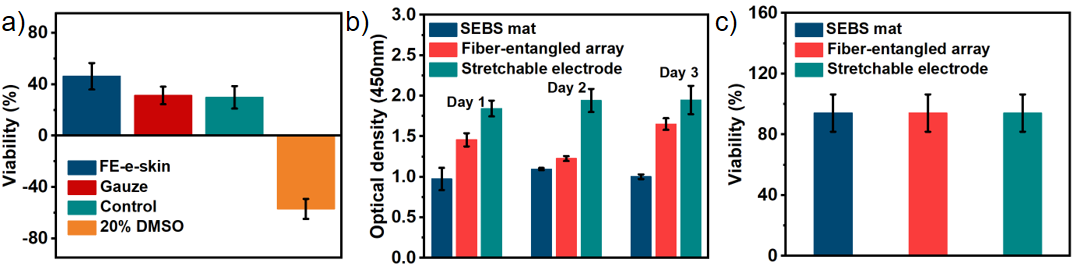


**Figure S24**. **Low cytotoxicity of the FE-e-skin. a)** Quantification of 4T1 mouse mammary cancer cells viability in different incubation groups including FE-e-skin, gauze, control, and 20% DMSO. **b)** Absorption at 450 nm in CCK-8 assay of different incubation groups after 1, 2 and 3 days of incubation. **c)** Quantification of 4T1 mouse mammary cancer cells viability in different incubation groups including SEBS mat, fiber-entangled array, and stretchable electrode.


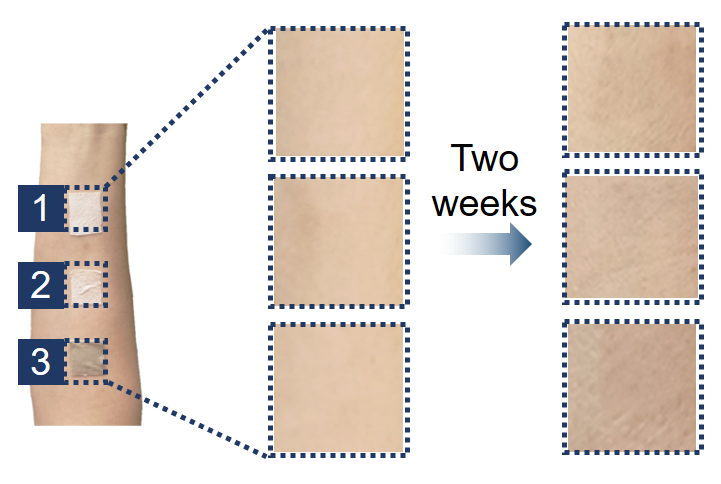


**Figure S25**. **High biocompatibility of the FE-e-skin.** Digital images showing the skin irritation results of different component layers on the forearms of the volunteer. 1: SEBS mat substrate; 2: Fiber-entangled island array; 3: Stretchable electrode array.


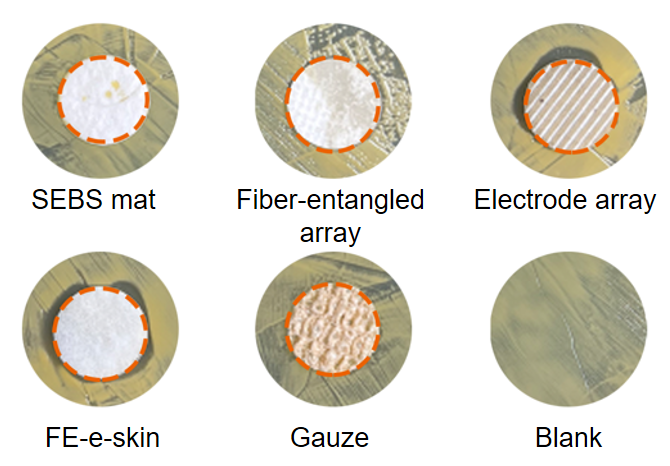


**Figure S26**. **Antibacterial property of the FE-e-skin.** Antibacterial assays conducted with Staphylococcus aureus demonstrated that the electrode array and FE-e-skin formed distinct inhibition zones, whereas the SEBS mat, fiber-entangled array, and gauze exhibited no detectable antibacterial activity. These results suggest that the antimicrobial efficacy of FE-e-skin is likely attributable to Ag nanoparticle integrated within the electrode array.

**Figure S27**. **High-throughput capacitance acquisition by integrated circuit. a)** The FE-e-skin was connected to the readout circuitry via FFCs. **b)** Photograph showing the appearance of the readout circuitry. The readout circuitry incorporates an SPDT array and a CDC array, with control implemented via an FPGA.


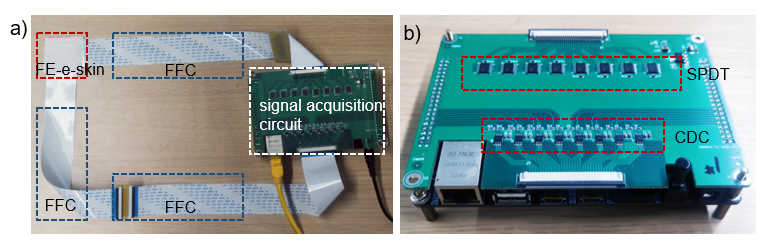


**Figure S28**. **Imaging and post-processing of data.** The hexadecimal arrays transmitted from the FPGA were converted to decimal format, subsequently transformed into capacitance variation rates, and arranged into a matrix, which was then imaged using Matlab. During the imaging process, a smoothing algorithm was applied to enhance the fidelity of the reconstructed images.


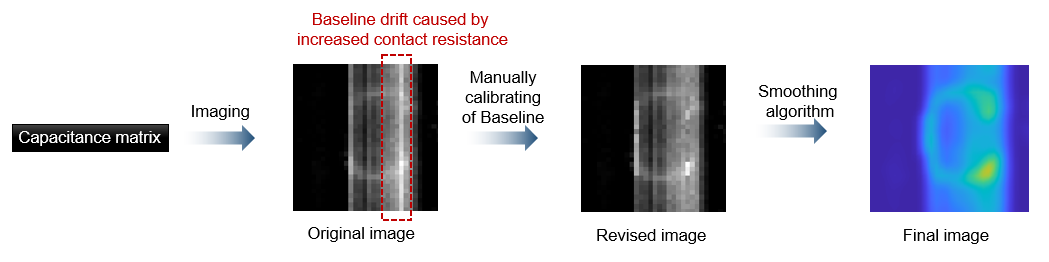

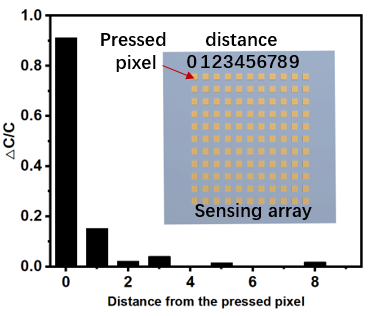


**Figure S29:** The relationship between capacitance change in a sensing array and the distance from the pressed pixel.


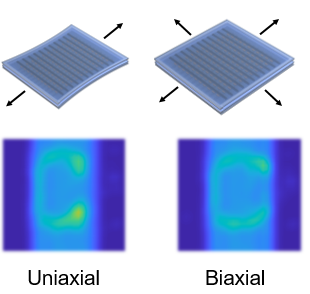


**Figure S30**. **Biaxial stretching of the FE-e-skin.** Besides uniaxial stretching, the FE-e-skin also possessed strain-insensitive pressure imaging performance under biaxial stretching.


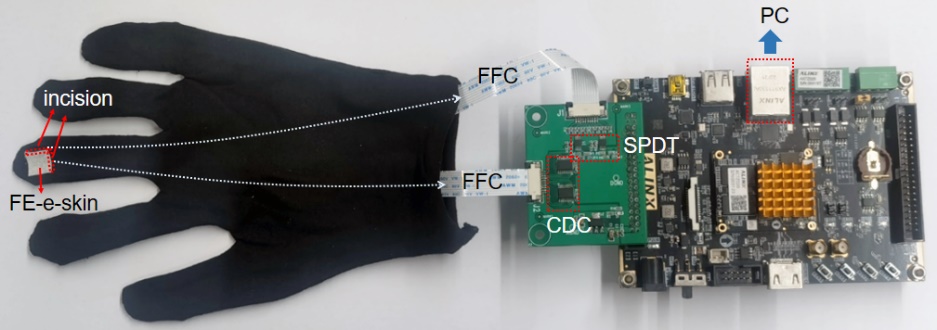


**Figure S31**. Photograph showing the appearance of the tactile glove, as well as the connection method between the readout circuitry and the tactile glove.


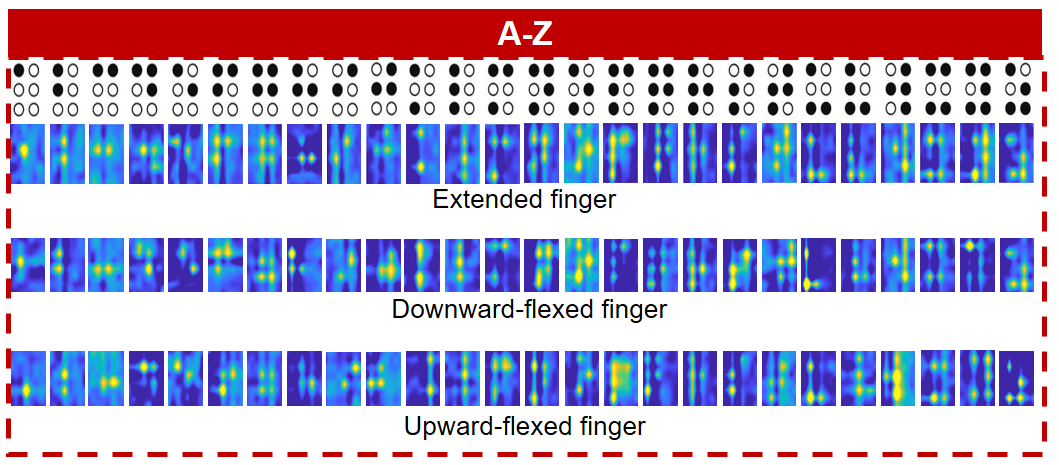


**Figure S32**. Pressure images of various Braille characters from A to Z captured under various gestures indicated that the images remained gesture-invariant.

**Figure S33**. Data augmentation of the original dataset resulted in a high-quality dataset comprising over 500000 images. Each image in the original dataset was first transformed into 10 images through random translation. Subsequently, each generated image was further enhanced with random contrast adjustments and Gaussian blur, producing 3 additional images per transformation to construct the augmented dataset.


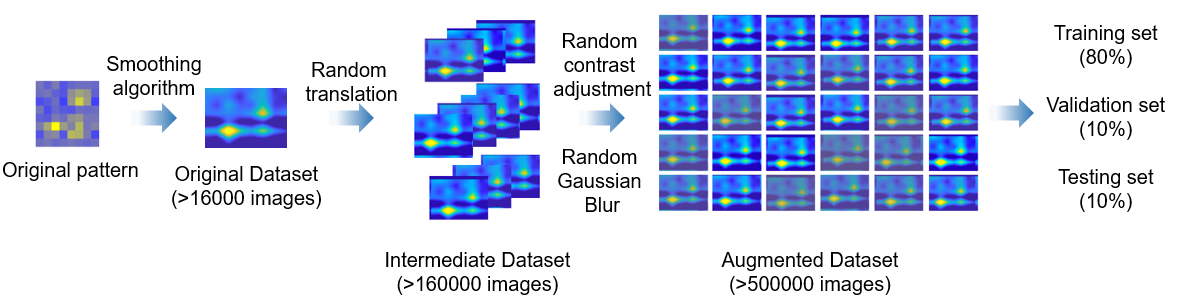

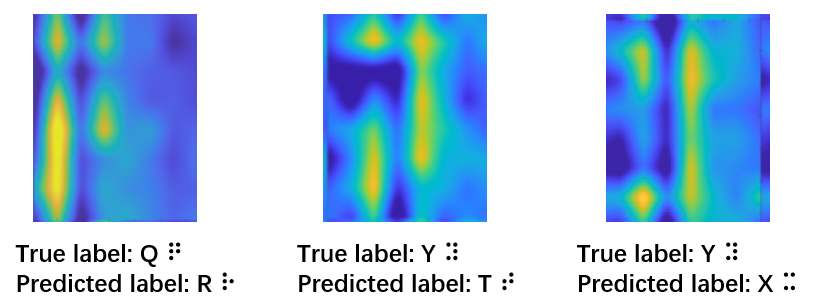


**Figure S34:** Representative Braille images that were misclassified in the confusion matrix.

**Additional discussion of Figure S34:**

Some Braille characters are highly similar and complex, leading them to be easily misidentified. For example, about 3.6% “Y (⠽)” characters were misclassified as “X (⠭)” and “T (⠞)”, and about 9% of “Q (⠟)” were misclassified as “R (⠗)”. Representative examples of these misidentified characters are shown in Figure R3.

This phenomenon can be optimized from two aspects:

1. **Data augmentation:** Additional data augmentation strategies, including targeted occlusion of discriminative regions in visually similar Braille characters, combined with increased weighting of confusable character pairs in the training set, could help the model to better learn the subtle differences between these characters.
2. **Model improvement:** Incorporating a lightweight attention module into the ResNet34 architecture can help the model better capture subtle differences in Braille characters. Alternatively, employing a deeper network may improve classification accuracy, particularly for highly similar characters.


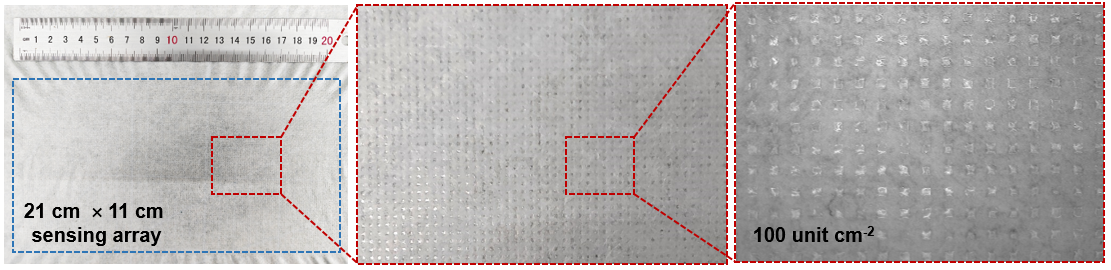


**Figure R8:**

**Figure S35:** The 21 cm  × 11 cm sensing array (100 unit cm^-2^) with high uniformity fabricated by a screen-printing machine.

**Additional discussion of Figure S35:**

To validate the feasibility of scalable fabrication, a screen-printing machine (Hefei Kejing Materials Technology Co. LTD., SPC-3050) was employed to fabricate a 21 cm  × 11 cm sensing array (100 unit cm^-2^) with high uniformity on a commercial TPU nonwoven membrane, as shown in Figure S35. By utilizing automated printing systems with a larger printing area, the fabrication process can be further scaled up, demonstrating the strong commercialization potential of this e-skin technology.

Although the demonstrated scalability highlights the commercialization potential of the sensing array, technical challenges remain to be addressed before practical deployment. A key issue is the unstable electrical contact between large-area sensing arrays and external circuits, which arises from the anisotropic conductive film (ACF) used to connect the devices to cables

Due to the limited thermal stability of the SEBS substrate, the hot-pressing temperature during ACF bonding process was restricted to 100°C, resulting in unreliable device-cable connections. Under applied pressure, the contact resistance between the device and cable increased, resulting in elevated capacitance measurements across all units in the affected column.

This issue could potentially be resolved by replacing the ACF with a biphasic, nano-dispersed (BIND) interface. The BIND interface represents a SEBS-compatible material that facilitates interconnection of densely packed electrode arrays (Ref. 54 in the manuscript). This innovative platform combines low-cost fabrication with straightforward processing, rendering it particularly advantageous for commercialization efforts.

|  | **0% strain** | **30% strain** | **Deviation** |
| --- | --- | --- | --- |
| **0 kPa** | 0 | -0.02 | \ |
| **1 kPa** | 0.3150 | 0.3131 | 0.63% |
| **5 kPa** | 1.7149 | 1.7341 | 1.12% |
| **10 kPa** | 2.2125 | 2.1691 | 1.9% |
| **50 kPa** | 3.4691 | 3.504 | 1% |

**Table S1. Low strain interference of the FE-e-skin.** The capacitance readings for different pressures applied with 0% strain and 30% strain vary by about 1% on average.

**Table S2. Performance comparison of the FE-e-skin and state-of-the-art pressure-sensing electronic skins.** The FE-e-skin represented the only device strategy to date that simultaneously achieves high pixel density, low strain interference, and high permeability.

| No. | GF | Pixel density  (unit cm^-2^) | Pixel number | Stretchability  (%) | Response time  (ms) | Permeability | | Ref |
| --- | --- | --- | --- | --- | --- | --- | --- | --- |
| 1 | 0.084 | 1 | 9 | 50 | 50 | × | 1 | |
| 2 | 3.2 | 1.78 | 25 | 40 | 31 | × | 2 | |
| 3 | 0.11 | 4 | 16 | 45 | 190 | × | 3 | |
| 4 | 0.21 | 0.12 | 9 | 60 | 50 | × | 4 | |
| 5 | 0.43 | 1 | 9 | 125 | 307 | × | 5 | |
| 6 | 3.3 | 36 | 100 | 1.2 | \ | × | 6 | |
| 7 | 0.26 | 5.53 | 64 | \ | 70 | × | 7 | |
| 8 | 2.52 | 2.77 | 36 | 50 | 330 | × | 8 | |
| 9 | 0.46 | 0.44 | 36 | 100 | 19.9 | × | 9 | |
| 10 | 2.5 | 1 | 8 | 140 | 6 | × | 10 | |
| 11 | 2.92 | 9 | 36 | 50 | 284 | × | 11 | |
| 12 | 0.2 | 1.96 | 49 | 50 | \ | × | 12 | |
| 13 | 0.22 | 0.64 | 16 | 30 | 170 | × | 13 | |
| 14 | 0.55 | 1 | 16 | 50 | \ | × | 14 | |
| 15 | 1.04 | 0.36 | 9 | 50 | \ | × | 15 | |
| 16 | unstretchable | 255 | 81 | 0 | 0.61 | × | 16 | |
| 17 | unstretchable | 1600 | 36 | 0 | 9 | × | 17 | |
| 18 | unstretchable | 8464 | 8464 | 0 | 150 | × | 18 | |
| 19 | unstretchable | 522 | 1024 | 0 | 34 | × | 19 | |
| 20 | unstretchable | 204 | 64 | 0 | 20 | × | 20 | |
| 21 | 0.25 | Single pixel | 1 | 30 | \ | × | 21 | |
| 22 | 0.17 | Single pixel | 1 | 30 | 20 | × | 22 | |
| 23 | \ | 0.48 | 1024 | \ | \ | √ | 23 | |
| 24 | 0.03 | 100 | 10000 | 30 | 12 | √ | This work | |
| 25 | 0 | 240 | 540 | 30 | 30 | √ | Human fingertip | |

**References**

1 Q. Su, Q. Zou, Y. Li, Y. Chen, S. Teng, J. T. Kelleher, R. Nith, P. Cheng, N. Li, W. Liu, S. Dai, Y. Liu, A. Mazursky, J. Xu, L. Jin, P. Lopes, S. Wang, A stretchable and strain-unperturbed pressure sensor for motion interference–free tactile monitoring on skins. *Sci. Adv.,* **2021***,* *7*, eabi4563.

2 Y. Cheng, R. Wang, H. Zhai, J. Sun, Stretchable electronic skin based on silver nanowire composite fiber electrodes for sensing pressure, proximity, and multidirectional strain. *Nanoscale,* **2017***,* *9*, 3834-3842.

3 Y. Zhang, S. Liu, Y. Miao, H. Yang, X. Chen, X. Xiao, Z. Jiang, X. Chen, B. Nie, J. Liu*.* Highly stretchable and sensitive pressure sensor array based on icicle-shaped liquid metal film electrodes. *ACS Appl. Mater. Interface,* **2020***,* *12*, 27961-27970.

4 J. C. Yang, J. Kim, J. Oh, S. Y. Kwon, J. Y. Sim, D. W. Kim, H. B. Choi, S. Park, Microstructured porous pyramid-based ultrahigh sensitive pressure sensor insensitive to strain and temperature. ACS Appl. Mater. Interface, **2019**, *11*, 19472-19480.

5 H. Qin, R. E. Owyeung, S. R. Sonkusale, M. J. Panzer. Highly stretchable and nonvolatile gelatin-supported deep eutectic solvent gel electrolyte-based ionic skins for strain and pressure sensing. *J. Mater. Chem. C,* **2019***,* *7*, 601-608.

6 Q. Hua, J. Sun, H. Liu, R. Bao, R. Yu, J. Zhai, C. Pan, Z. L. Wang, Skin-inspired highly stretchable and conformable matrix networks for multifunctional sensing. *Nat. Commun.,* **2018***,* *9*, 244.

7 X. Wang, Y. Zhang, X. Zhang, Z. Huo, X. Li, M. Que, Z. Peng, H. Wang, C. Pan*.* A highly stretchable transparent self‐powered triboelectric tactile sensor with metallized nanofibers for wearable electronics. *Adv. Mater.,* **2018***, 30*, 1706738.

8 M. Liu, X. Zhao, C. Hang, L. Zhu, X. Wu, X. Wen, J. Wang, H. Lu. A stretchable pressure sensor with interlinked interfaces prepared by a template-free process. *Compos. Part. A-Appl. S.,* **2022***,* *162*, 107144.

9 J. Park, Y. Ko, J. Y. Cho, S. Lee, Y. Lee, J. Han, H. Ko, Stretchable ionic composites for strain-insensitive dual-mode pressure and proximity sensors. *Chem. Eng. J.,* **2024***,* *480*, 148172.

10 Y. Zhang, J. Yang, X. Hou, G. Li, L. Wang, N. Bai, M. Cai, L. Zhao, Y. Wang, J. Zhang, K. Chen, X. Wu, C. Yang, Y. Dai, Z. Zhang, C. Guo, Highly stable flexible pressure sensors with a quasi-homogeneous composition and interlinked interfaces. *Nat. Common.,* **2022***,* *13*, 1317.

11 Y. Zhang, Q. Lu, J. He, Z. Huo, R. Zhou, X. Han, M. Jia, C. Pan, Z. Wang, J. Zhai, Localizing strain via micro-cage structure for stretchable pressure sensor arrays with ultralow spatial crosstalk. *Nat. Commun.,* **2023***,* *14*, 1252.

12 X. Zhao, Q. Hua, R. Yu, Y. Zhang, C. Pan. Flexible, stretchable and wearable multifunctional sensor array as artificial electronic skin for static and dynamic strain mapping. *Adv. Electron. Mater.,* **2015***,* *1*, 1500142.

13 C. Zhang, S. Liu, X. Huang, W. Guo, Y. Li, H. Wu*.* A stretchable dual-mode sensor array for multifunctional robotic electronic skin. *Nano Energy,* ***2019****, 62*, 164-170.

14 S.-J. Woo, J.-H. Kong, D.-G. Kim, J.-M. Kim, A thin all-elastomeric capacitive pressure sensor array based on micro-contact printed elastic conductors. *J. Mater. Chem. C,* **2014***,* *2*, 4415-4422.

15 R. Matsuda, S. Mizuguchi, F. Nakamura, T. Endo, Y. lsoda, G. lnamori, H. Ota, Highly stretchable sensing array for independent detection of pressure and strain exploiting structural and resistive control. *Sci. Rep.,* **2020***,* *10*, 12666.

16 N. Bai, L. Wang, Y. Xue, Y. Wang, X. Hou, G. Li, Y. Zhang, M. Cai, L. Zhao, F. Guan, X. Wei, C. Guo, Graded interlocks for iontronic pressure sensors with high sensitivity and high linearity over a broad range. *ACS Nano,* **2022***,* *16*, 4338-4347.

17 N. Bai, L. Wang, Q. Wang, J. Deng, Y. Wang, P. Lu, J. Huang, G. Li, Y. Zhang, J. Yang, K. Xie, X. Zhao, C. Guo, Graded intrafillable architecture-based iontronic pressure sensor with ultra-broad-range high sensitivity. *Nat. Commun.,* **2020***,* *11*, 209.

18 W. Wu, X. Wen, Z. L. Wang, Taxel-addressable matrix of vertical-nanowire piezotronic transistors for active and adaptive tactile imaging. *Science,* **2013***,* *340*, 952-957.

19 Y. Cheng, C. Guo, S. Li, K. Deng, J. Tang, Q. Luo, S. Zhang, Y. Chang, T. Pan, Aquatic skin enabled by multi‐modality iontronic sensing. *Adv. Funct. Mater.,* **2022***,* *32*, 2205947.

20 Y. Li, J. Long, Y. Chen, Y. Huang, N. J. A. M. Zhao, Crosstalk‐free, high‐resolution pressure sensor arrays enabled by high‐throughput laser manufacturing. *Adv. Mater.,* **2022***,* *34*, 2200517.

21 J. Yang, Y. Xu, Q. Guo, F. Yin, W. Yuan. Highly stretchable pressure sensors with wrinkled fibrous geometry for selective pressure sensing with minimal lateral strain-induced interference. *Compos. Part. B-Eng.,* **2021***,* *217*, 108899.

22 E. Roh, H. Lee, D. Kim, N. Lee. A solution‐processable, omnidirectionally stretchable, and high‐pressure‐sensitive piezoresistive device. *Adv. Mater.,* **2017***,* *29*, 1703004.

23 Y. Luo, Y. Li, P. Sharma, W. Shou, T. Palacios, A. Torralba, W. Matusik, Learning human-environment interactions using conformal tactile textiles. *Nat. Electron.,* **2021***,* *4*, 193-201.
